# Supplementary material for: Characterization and bioactivity potential of marine sponges (Biemna fistulosa, Callyspongia diffusa, and Haliclona fascigera) from Kenyan coastal waters
Source: PLoS One. 2025 Jul 24;20(7):e0325642. doi: 10.1371/journal.pone.0325642 (PMC12289071; doi:10.1371/journal.pone.0325642)
Supplement: S1 Table — (PDF) [file pone.0325642.s001.pdf]

Manuscript: PONE-D-25-26894

Supporting information

S1 Table: Summary of marine sponge species observed in high abundance (≥4 sites) across Kenyan coastal study areas

Sponge Species (Taxon)	Morphotype / Color	Sites	Habitat Notes
Carteriospongia foliascens	Fan-shaped / Green	Kuruwitu, Kanamai, Sii Island, Mtwapa Creek, Mundini and Ras Kiromo	Found on mangroves, sandy lagoons, seagrass beds, sandy beaches, and coral reefs
Callyspongia diffusa	Tube-like / Brown		Common in seagrass beds and coral reef slopes; tolerates turbid shallow waters
Callyspongia plicifera	Tube-like / Orange		Often found on coral rubble and protected reef flats
Callyspongia siphonella	Tube-like / Cream		Typically occurs in shallow reef lagoons and sheltered bays
Haliclona tubifera	Encrusting / Green		Commonly associated with mangrove roots and soft-bottom habitats
Haliclona oculata	Encrusting / Brown		Attached to rocks and stones on sandy or rocky substrate
