# Supplementary material for: Characterization and bioactivity potential of marine sponges (Biemna fistulosa, Callyspongia diffusa, and Haliclona fascigera) from Kenyan coastal waters
Source: PLoS One. 2025 Jul 24;20(7):e0325642. doi: 10.1371/journal.pone.0325642 (PMC12289071; doi:10.1371/journal.pone.0325642)
Supplement: S2 Table — (PDF) [file pone.0325642.s002.pdf]

Manuscript: PONE-D-25-26894

Supporting information

S2 Table: Summary of marine sponge species recorded at moderate abundance (2–3 sites) along the Kenyan coastline

Sponge Species (Taxon)	Morphotype / Color	Sites	Habitat Notes
Biemna fistulosa	Encrusting / Brown	Ras Kiromo and Mtwapa Creek	Present along sandy shores and in mangrove lagoons
Tedania charcoti	Pear-shaped / Brown-Orange	Sii Island, Mtwapa Creek and Ras Kiromo	Shallow tropical waters, near mangrove roots and sandy lagoons
Callyspongia ramosa	Tube-like / Orange-Grey	Kanamai and Mtwapa Creek	Grows on reef crests and within shallow lagoons
Callyspongia pseudotoxa	Tube-like / Cream-Green	Kuruwitu, Kanamai and Mtwapa Creek	Found on sandy substrates and patch reef systems
Haliclona implexiformis	Encrusting / Purple		Typically associated with mangrove channels or soft-sediment substrates
Haliclona stilensis	Encrusting / Brown	Kuruwitu, Mtwapa Creek and Sii Island	Usually encrusts seagrass blades, rubble, or coral fragments
Haliclona cinerea	Encrusting / Purple	Kuruwitu, Kanamai, Ras Kiromo	Inhabits intertidal rock pools and coral reef flats
Callyspongia fallax	Tube-like / Grey	Mtwapa Creek, Mundini	Found on shallow sandy bottoms and in mangrove lagoons
Haliclona laubenfelsi	Encrusting / Cream-Green	Sii Island, Mundini, Ras Kiromo	Found in protected lagoons and among coral assemblages
Clathria rugosa	Fan-shaped / Brown		Grows on coral reef slopes and rubble zones
Clathria reinwardti	Fan-shaped / Red		Occurs in reef crevices and shaded lagoon habitats
Clathria prolifera	Fan-shaped / Orange		Found on rocky reef faces and in tide pools
Clathria parthena	Fan-shaped / Brown		Typically inhabits shallow coral reefs and rubble beds
