# Supplementary material for: Characterization and bioactivity potential of marine sponges (Biemna fistulosa, Callyspongia diffusa, and Haliclona fascigera) from Kenyan coastal waters
Source: PLoS One. 2025 Jul 24;20(7):e0325642. doi: 10.1371/journal.pone.0325642 (PMC12289071; doi:10.1371/journal.pone.0325642)
Supplement: S3 Table — (PDF) [file pone.0325642.s003.pdf]

Manuscript: PONE-D-25-26894

Supporting information

S3 Table: Summary of marine sponge species observed at rare abundance (single site) along the Kenyan coastline

**Sponge Species (Taxon)**	**Morphotype / Color**	**Sites**	**Habitat Notes**
Stylissa carteri	Fan-shaped / Orange	Sii Island	Found in mangrove lagoons, coral reef margins, and sandy beach zones
Agelas cerebrum	Tube-like / Brown	Mtwapa Creek	Typically found in cryptic reef environments, attached to hard substrata
Haliclona fascigera	Tube-like / Blue	Ras Kiromo	Shallow sandy bottoms of lagoons and coral reef vicinities
Paratetilla arcifera	Pear-shaped / Brown	Kanamai	Inhabits seagrass beds, shallow sandy lagoons, and sandy beaches
