# Supplementary material for: Characterization and bioactivity potential of marine sponges (Biemna fistulosa, Callyspongia diffusa, and Haliclona fascigera) from Kenyan coastal waters
Source: PLoS One. 2025 Jul 24;20(7):e0325642. doi: 10.1371/journal.pone.0325642 (PMC12289071; doi:10.1371/journal.pone.0325642)
Supplement: Attachment — Submitted filename: PONE-D-25-26894.pdf [file pone.0325642.s004.pdf]

PLOS ONE

Characterization and Bioactivity Potential of Marine Sponges (Biemna fistulosa, Callyspongia diffusa, and Haliclona fascigera) from Kenyan Coastal Waters

--Manuscript Draft--

**Manuscript Number:**	PONE-D-25-26894
**Article Type:**	Research Article
**Full Title:**	Characterization and Bioactivity Potential of Marine Sponges (Biemna fistulosa, Callyspongia diffusa, and Haliclona fascigera) from Kenyan Coastal Waters
**Short Title:**	Characterization and Bioactivity Potential of Marine Sponges
**Corresponding Author:**	TERESIA NYAMBURA WACIRA, PhD<br>Kenya Marine and Fisheries Research Institute<br>Kisumu, No State KENYA
**Keywords:**	Chemical compounds; COI gene; GC-MS chromatogram; Standard antibiotics
**Abstract:**	Sponges have been reported as a rich source of bioactive compounds, which could potentially be developed into lead compounds for pharmaceutical use. The present study aimed to identify sponges and assess the biological activity of their extracts against human disease-causing organisms, including Escherichia coli, Pseudomonas aeruginosa, Staphylococcus aureus, and Candida albicans. Morphological characterization and DNA barcoding of the cytochrome c oxidase subunit I (COI) gene characterized three sponge species (Biemna fistulosa, Callyspongia diffusa and Haliclona fascigera). The Kirby-Bauer test assessed the antimicrobial activity of the extracts and the inhibition zone diameters (IZD) were measured. The extracts were further subjected to minimum inhibitory concentration (MIC) and minimum bactericidal concentration (MBC) tests to determine the antibiotic susceptibility. The Gas Chromatography-Mass Spectrometry (GC-MS) was used to identify and quantify the organic compounds in the sponges' extracts. The methanolic extract of B. fistulosa (28.00 ± 3.5 mm) and H. fascigera. (28.33 ± 3.8 mm) exhibited a broad spectrum of antibacterial activity against E. coli surpassing the positive control (27.67 ± 0.9 mm). The inhibitory activity of ethyl acetate extract of the C. diffusa (29.33 ± 2.4 mm) against P. aeruginosa was observed to be higher compared to the standard antibiotic streptomycin (26.67±0.7 mm). The methanolic extract of H. fascigera demonstrated the lowest MIC (0.53 ± 0.0 mg/ml) in compared to the streptomycin drug (1.36 ± 0.0 mg/ml), and showed an MBC of 1.25 mg/ml against E. coli. The GC-MS chromatogram data analysis identified 114 distinct compounds categorized into 39 classes across three sponge extracts, with 11.4% of these compounds demonstrating documented antimicrobial activity against human pathogens. This study corroborates sponges as a promising source of bioactive compounds, which are valuable leads for drug discovery and development. Future research must explore their mechanisms, molecular-level toxicity, and lead optimization to enhance drug development.
**Order of Authors:**	TERESIA NYAMBURA WACIRA, PhD
	Huxley Mae Makonde, Ph.D
	Joseph Nyingi Kamau, Ph.D
	Christopher Mulanda Aura, Ph.D
	Cromwell Mwiti Kibiti, Ph.D
**Additional Information:**	
**Question**	**Response**
**Financial Disclosure**<br><br>Enter a financial disclosure statement that describes the sources of funding for the work included in this submission. Review	Yes

the submission guidelines for detailed requirements. View published research articles from *PLOS ONE* for specific examples.<br><br>This statement is required for submission and **will appear in the published article** if the submission is accepted. Please make sure it is accurate.<br><br>**Funded studies**<br>Enter a statement with the following details:<br>• Initials of the authors who received each award<br>• Grant numbers awarded to each author<br>• The full name of each funder<br>• URL of each funder website<br>• Did the sponsors or funders play any role in the study design, data collection and analysis, decision to publish, or preparation of the manuscript?<br><br>Did you receive funding for this work?	
Please add funding details.<br>as follow-up to "**Financial Disclosure**<br><br>Enter a financial disclosure statement that describes the sources of funding for the work included in this submission. Review the submission guidelines for detailed requirements. View published research articles from *PLOS ONE* for specific examples.<br><br>This statement is required for submission and **will appear in the published article** if the submission is accepted. Please make sure it is accurate.	We sincerely thank the Kenya Marine and Fisheries Research Institute (KMFRI) for their invaluable support in partially funding for the research (GOK-PC Target C82 39-1).

**Funded studies**<br>Enter a statement with the following details:<br>• Initials of the authors who received each award<br>• Grant numbers awarded to each author<br>• The full name of each funder<br>• URL of each funder website<br>• Did the sponsors or funders play any role in the study design, data collection and analysis, decision to publish, or preparation of the manuscript?<br><br>Did you receive funding for this work?"	
Please select the country of your main research funder (please select carefully as in some cases this is used in fee calculation).<br>as follow-up to "**Financial Disclosure**<br><br>Enter a financial disclosure statement that describes the sources of funding for the work included in this submission. Review the submission guidelines for detailed requirements. View published research articles from *PLOS ONE* for specific examples.<br><br>This statement is required for submission and **will appear in the published article** if the submission is accepted. Please make sure it is accurate.<br><br>**Funded studies**<br>Enter a statement with the following details:<br>• Initials of the authors who received each award<br>• Grant numbers awarded to each author<br>• The full name of each funder<br>• URL of each funder website<br>• Did the sponsors or funders play any role in the study design, data collection and analysis, decision to publish, or preparation of the manuscript?<br><br>Did you receive funding for this work?"	KENYA - KE
**Competing Interests**	The authors have declared that no competing interests exist.

Use the instructions below to enter a competing interest statement for this submission. On behalf of all authors, disclose any competing interests that could be perceived to bias this work—acknowledging all financial support and any other relevant financial or non-financial competing interests.<br><br>This statement is required for submission and **will appear in the published article** if the submission is accepted. Please make sure it is accurate and that any funding sources listed in your Funding Information later in the submission form are also declared in your Financial Disclosure statement.<br><br>View published research articles from *PLOS ONE* for specific examples.<br><br>**NO authors have competing interests**<br><br>Enter: *The authors have declared that no competing interests exist.*<br><br>**Authors with competing interests**<br><br>Enter competing interest details beginning with this statement:<br><br>*I have read the journal's policy and the authors of this manuscript have the following competing interests: [insert competing interests here]*<br><br>* typeset	
**Ethics Statement**<br><br>Enter an ethics statement for this submission. This statement is required if the study involved:<br><br>• Human participants<br>• Human specimens or tissue<br>• Vertebrate animals or cephalopods<br>• Vertebrate embryos or tissues	PC Target C82 39-1-KENYA MARINE AND FISHERIES RESEARCH INSTITUTE

• Field research<br><br>Write "N/A" if the submission does not require an ethics statement.<br><br>General guidance is provided below. Consult the submission guidelines for detailed instructions. **Make sure that all information entered here is included in the Methods section of the manuscript.**	

Powered by Editorial Manager® and ProduXion Manager® from Aries Systems Corporation

**Format for specific study types**<br><br>**Human Subject Research (involving human participants and/or tissue)**<br>• Give the name of the institutional review board or ethics committee that approved the study<br>• Include the approval number and/or a statement indicating approval of this research<br>• Indicate the form of consent obtained (written/oral) or the reason that consent was not obtained (e.g. the data were analyzed anonymously)<br><br>**Animal Research (involving vertebrate animals, embryos or tissues)**<br>• Provide the name of the Institutional Animal Care and Use Committee (IACUC) or other relevant ethics board that reviewed the study protocol, and indicate whether they approved this research or granted a formal waiver of ethical approval<br>• Include an approval number if one was obtained<br>• If the study involved *non-human primates*, add *additional details* about animal welfare and steps taken to ameliorate suffering<br>• If anesthesia, euthanasia, or any kind of animal sacrifice is part of the study, include briefly which substances and/or methods were applied<br><br>**Field Research**<br><br>Include the following details if this study involves the collection of plant, animal, or other materials from a natural setting:<br>• Field permit number<br>• Name of the institution or relevant body that granted permission	
**Data Availability**<br><br>Authors are required to make all data underlying the findings described fully available, without restriction, and from the time of publication. PLOS allows rare exceptions to address legal and ethical concerns. See the PLOS Data Policy and FAQ for detailed information.	Yes - all data are fully available without restriction

A Data Availability Statement describing where the data can be found is required at submission. Your answers to this question constitute the Data Availability Statement and **will be published in the article**, if accepted.<br><br>**Important:** Stating 'data available on request from the author' is not sufficient. If your data are only available upon request, select 'No' for the first question and explain your exceptional situation in the text box.<br><br>Do the authors confirm that all data underlying the findings described in their manuscript are fully available without restriction?	
**Describe where the data may be found in full sentences. If you are copying our sample text, replace any instances of XXX with the appropriate details.**<br><br>• If the data are **held or will be held in a public repository**, include URLs, accession numbers or DOIs. If this information will only be available after acceptance, indicate this by ticking the box below. For example: *All XXX files are available from the XXX database (accession number(s) XXX, XXX.)*.<br>• If the data are all contained **within the manuscript and/or Supporting Information files**, enter the following: *All relevant data are within the manuscript and its Supporting Information files.*<br>• If neither of these applies but you are able to provide **details of access elsewhere**, with or without limitations, please do so. For example:<br><br>*Data cannot be shared publicly because of [XXX]. Data are available from the XXX Institutional Data Access / Ethics Committee (contact via XXX) for researchers who meet the criteria for access to confidential data.*<br><br>*The data underlying the results presented in the study are available from (include the name of the third party*	All relevant data are within the manuscript and its Supporting Information files.

and contact information or URL).<br>• This text is appropriate if the data are owned by a third party and authors do not have permission to share the data.<br><br>* typeset	
Additional data availability information:	

manuscript
Click here to access/download;Manuscript;Wacira_manuscript_2025.docx

Characterization and Bioactivity Potential of Marine Sponges (*Biemna fistulosa, Callyspongia diffusa,* and *Haliclona fascigera*) from Kenyan Coastal Waters

Teresia Nyambura Wacira[1,3*], Huxley Mae Makonde[1], Joseph Kamau Nyingi[2], Christopher Mulanda Aura[3], Cromwell Mwiti Kibiti[1]

[1]Department of Pure and Applied Sciences, Technical University of Mombasa, P. O. Box 90420-80100, Mombasa, Kenya.

[2]Kenya Marine and Fisheries Research Institute, P. O. Box 81651-80100, Mombasa, Kenya.

[3]Kenya Marine and Fisheries Research Institute, Freshwater Research Center, P.O. Box 1881, Kisumu, Kenya.

Corresponding Author*

Teresia Nyambura Wacira

Email: tnyambura@kmfri.go.ke; Phone number: +254721723213

Huxley M. Makonde: Email: makondehuxley@tum.ac.ke: Phone number: +254725535161

Joseph K. Nyingi: Email: jkamau@kmfri.go.ke: Phone number: +254718936598

Christopher M. Aura: Email: chrisaura@kmfri.go.ke: Phone number: +254711233774

Cromwell M. Kibiti: Email: cromwellkibiti@tum.ac.ke: Phone number: +254791427415

ABSTRACT

Sponges have been reported as a rich source of bioactive compounds, which could potentially be developed into lead compounds for pharmaceutical use. The present study aimed to identify sponges and assess the biological activity of their extracts against human disease-causing organisms, including *Escherichia coli*, *Pseudomonas aeruginosa, Staphylococcus aureus,* and *Candida albicans*. Morphological characterization and DNA barcoding of the cytochrome c oxidase subunit I (COI) gene characterized three sponge species (*Biemna fistulosa*, *Callyspongia diffusa* and *Haliclona fascigera*). The Kirby-Bauer test assessed the antimicrobial activity of the extracts and the inhibition zone diameters (IZD) were measured. The extracts were further subjected to minimum inhibitory concentration (MIC) and minimum bactericidal concentration (MBC) tests to determine the antibiotic susceptibility. The Gas Chromatography-Mass Spectrometry (GC-MS) was used to identify and quantify the organic compounds in the sponges' extracts. The methanolic extract of *B. fistulosa* (28.00 ± 3.5 mm) and *H. fascigera*. (28.33 ± 3.8 mm) exhibited a broad spectrum of antibacterial activity against *E. coli* surpassing the positive control (27.67 ± 0.9 mm). The inhibitory activity of ethyl acetate extract of the *C. diffusa* (29.33 ± 2.4 mm) against *P. aeruginosa* was observed to be higher compared to the standard antibiotic streptomycin (26.67±0.7 mm). The methanolic extract of *H. fascigera* demonstrated the lowest MIC (0.53 ± 0.0 mg/ml) in compared to the streptomycin drug (1.36 ± 0.0 mg/ml), and showed an MBC of 1.25 mg/ml against *E. co*[illegible] The GC-MS chromatogram data analysis identified 114 distinct compounds categorized into 39 classes across three sponge extracts, with 11.4% of these compounds demonstrating documented antimicrobial activity against human pathogens. This study corroborates sponges as a promising source of bioactive compounds, which are valuable leads for drug discovery and development. Future research must explore their mechanisms, molecular-level toxicity, and lead optimization to enhance drug development.

Keywords: Chemical compounds, COI gene, GC-MS chromatogram, Standard antibiotics

Introduction

Despite a record dating back at least the entire Phanerozoic, approximately 600 million years (Carrier et al., 2022). The magnitude of aquatic marine sponge biodiversity is unknown (Folkers and Rombouts, 2020). As of March 2025, the World Porifera Database (WPD) (Soest et al., 2019) recognizes approximately 9,722 accepted living sponge species and 60 extinct species. In 1950, Bergmann and Feeney discovered marine sponge natural products by extracting novel spongothymidine and spongouridine nucleosides from *Tectitethya crypta*, formerly known as *Cryptotheca crypta* (Kurian and Elumalai, 2017). Among the three classes of sponges, class Demospongiae and orders Halichondrida, Poecilosclerida, and Dictyoceratida are the significant sources of bioactive compounds compared to Hexactinellida and Calcarea (Sharma et al., 2010). Recent scholarly investigations underscore the considerable potential of marine sponges as a source for novel antimicrobial agents. Notably, a study conducted on sponges from Saint Thomas, U.S. Virgin Islands, demonstrated significant antibacterial activity against several pathogenic microorganisms, including *Escherichia coli*, *Staphylococcus aureus*, and *Neisseria gonorrhoeae* (Christian et al., 2024). More than 200 new compounds have been isolated from marine sponges, accounting for approximately 23% of approved marine-derived pharmaceuticals (Hong et al., 2022).

Dragmacidin G, a bioactive alkaloid extracted from sponges of the genera *Spongosorites* and *Lipastrotheya*, exhibited a broad spectrum of biological activity, including the inhibition of *Mycobacterium tuberculosis*, *Plasmodium falciparum* and methicillin-resistant *Staphylococcus*

aureus (MRSA) (Wright et al., 2017). Manzamine A, derived from Indo-Pacific sponges, manifests broad-spectrum antibacterial activity against both Gram-positive and Gram-negative bacterial strains (Santi et al., 2025). Similarly, Aeroplysinin-1, extracted from *Aplysina aerophoba*, has revealed significant antibacterial properties against various pathogenic strains (García-Vilas et al., 2016). Additionally, compounds such as Discorhabdin G and Ageliferin from sponges have been recognized for their ability to prevent bacterial biofilm formation, a critical factor that contributes to the persistence and resilience of infections (Huigens, 2009). Furthermore, the pharmacological potential of marine sponges transcends antibacterial activity, as they are also abundant sources of antifungal and antiviral agents. Avarol, a hydroquinone derivative isolated from the sponge *Dysidea avara*, has antifungal activity against various pathogenic fungi (Esposito et al., 2022). Mycalamide A, obtained from *Mycale* sp., is renowned for its antiviral activity, particularly against herpes simplex virus and poliovirus type 1 (Sharma et al., 2016).

Despite the significant advancements made in the research of marine sponges for the production of bioactive compounds, considerable gaps remain in our understanding of marine sponges and their bioactive compounds (Mehbub et al., 2024). Furthermore, while various sponge-derived compounds are recognized for their antibacterial and antifungal activities, there is inadequate literature and comprehensive data on the identification and bioactivity studies of marine sponges from the Kenyan coastal waters.

(Handayani et al., 2023). This scientific gap impedes the progression of sponge bioactive compounds from laboratory studies to clinical applications, a situation exacerbated by the scarcity of high-throughput screening technologies (Ayon, 2023). Numerous coastal and deep-sea sponge species, particularly those inhabiting African waters, such as those off the coast of Kenya, remain

largely underexplored (Garritano et al., 2023). To bridge the scientific gap, the present comprehensive study identified some marine sponge species and characterized their antimicrobial potential against selected human pathogens.

MATERIALS AND METHODS

Description of study site

The study sites included Sii Island (4°40'46.1"S, 39°17'01.9"E), Mundini (4°39'10.0"S, 39°21'41.0" E), and Ras Kiromo (4°38'45.5"S, 39°19'23.4"E), located on the South Coastline of Kenya. These are sites characterized by diverse marine ecosystems, with coral reefs and seagrass beds supporting a wide range of aquatic organisms (Musembi et al., 2019).

Metazoan specimen collection and preparation

A pre-survey for marine sponge sampling was undertaken to determine optimal conditions for clear underwater photography and effective sampling during favorable weather conditions (Cai et al., 2024). The collection of metazoan marine sponges took place during the low spring tide, coinciding with the southeast monsoon in September 2022 (Maldonado et al., 2012). The sponge specimens were collected from depths of 3 to 5 meters using snorkeling and SCUBA diving techniques (Cleary et al., 2023). At each site, the sponges were sampled based on the purposive sampling criteria and subsequently rinsed with sterile ocean water to remove debris and epiphytic organisms (Williams et al., 2020).

Specimens were placed in labeled containers filled with sterile ocean water and transported to the Kenya Marine and Fisheries Research Institute (KMFRI) laboratories. Preservation methods were

selected based on the intended further analyses: samples were frozen at -20°C for genetic studies and antimicrobial activity assessment, and preserved in 70% ethanol for morphological evaluations (Schöttner et al., 2013).

Morphological characterization of the marine sponges

The sponge morphological identification relied on external morphology, encompassing the color, shape, and surface features (Hadi et al., 2016). The spicules and spongin fibers within the marine sponge skeleton were digested into small pieces of sponge tissue in bleach and observed under a Primo Star ZEISS image analyzer microscope with a coverslip. The microphotographs were taken using an Axio cam ERc5s digital camera (Carl Zeiss, Germany) (Jensen et al., 2011). The marine sponge specimens voucher numbers: BLSi 007, BRMu 004, and BLD 014 used in this study are preserved at the Kenya Marine and Fisheries Research Institute Museum. The marine sponge species was determined using the World Register of Marine Species (WoRMS) database (Costello et al., 2013). To identify the genus and species of marine sponges, the Sponge Identification Reference Book and the Porifera database list (http://www.marinespecies.org/porifera/) were employed (Sandes et al., 2024).

DNA Extraction and PCR Amplification of the Mitochondrial Gene Cytochrome c Oxidase Subunit I (COI)

The salting out method was used for DNA extraction for the sponge specimens (Daniels et al., 2023). First, 200 µl of the extraction buffer and 5 µl of proteinase K (Thermo Fisher Scientific, Unites States) were added to the tissue. The mixture was incubated at 37°C overnight. Following the incubation, the proteinase K was added, and the mixture was spun down, and 450 µl of 3M

sodium chloride (NaCl) was added and vortexed (Goldschmidt et al., 2014). The sample was then centrifuged at a speed of ≥10,000 g for 15 minutes to collect cell debris. The supernatant, containing DNA (1 ml), was transferred to a sterile 1.5 ml tube and mixed with frozen (-20°C) absolute ethanol to fill the tube (Marín et al., 2021). The DNA was allowed to precipitate overnight in a freezer at -20°C, followed by centrifugation at a speed of ≥10,000 g for 20 minutes. The pellet was washed thrice with 700 µl of 70% ethanol and then centrifuged at a speed of ≥10,000 g for another 20 minutes. The ethanol was pipetted out without disturbing the pellet, which was allowed to dry at room temperature (Holub et al., 2022). The DNA was then resuspended in 50 µl of deionized water (molecular grade) (Carl Roth, Germany) and its concentration and purity were assessed using a NanoDrop spectrophotometer (Thermo Fisher Scientific, United States).

The degenerate universal barcoding primers dgLCO1490 (GGT CAA CAA ATC ATA AAG AYA TYG G) and dgHCO2198 (TAA ACT TCA GGG TGA CCA AAR AAY CA), targeting the mitochondrial gene cytochrome c oxidase subunit I (COI) were used for amplification (Patantis et al., 2014). The PCR reaction mixture of 25 µL was prepared by mixing 12.5 µL of 2X PCR Master Mix (DreamTaq Green PCR, Thermo Fisher Scientific, United States), which included Taq polymerase, dNTPs, and buffer. Additionally, 1.0 µL of both the forward and reverse primers (10 µM) and 2.0 µL of template DNA (20 ng/µL) were added. The reaction volume was then brought to 25.0 µL by adding 8.5 µL of nuclease-free water. For PCR product verification, a 1.5% agarose gel (Sigma-Aldrich, Merck Millipore) was prepared in 1X TAE buffer, and stain G (SERVA Electrophoresis, Germany) was added (Timmers et al., 2022). A 5 µL aliquot of the PCR product was mixed with 1 µL of SYBR Green dye (Thermo Fisher Scientific, United States) and loaded into the gel wells. Distilled water was used as the negative control, while the DNA ladder (CSL-

MDNA, Cleaver Scientific) was used as a DNA marker. The gel was run at 100V for 30 minutes, and the PCR products were visualized under UV light using a gel documentation system (ATTO, Tokyo, Japan).

The PCR products were treated with an ExoSAP treatment (Kanakaraj et al., 2011). The purified products were then shipped to Inqaba Biotec, a commercial sequencing service provider in South Africa, for Sanger sequencing.

DNA Sequencing and Phylogenetic Analysis

Sequencing was conducted using the same primers (dgLCO1490 and dgHCO2198) that were used for PCR amplification. The reaction mixture consisted of the purified DNA template, sequencing primers, BigDye Terminator v3.1 Cycle Sequencing Kit, and buffer (SenGupta and Cookson, 2010). The prepared reaction mixture was subjected to cycle sequencing and then loaded onto an automated DNA sequencer (Applied Biosystems 3500XL Genetic Analyzer). The fluorescence data were interpreted using the FinchTV analysis software to generate the DNA sequence (Shuhaib and Hashim, 2023).

The raw sequence data from Sanger sequencing were processed using BioEdit software version 7.2. Low-quality bases were identified and removed to maintain data integrity. The sequences were compared against the nucleotide sequence database (GenBank) at the National Center for Biotechnology Information (NCBI) and Barcode of Life Database (BOLD) using the BLASTn algorithm to determine species identity. Additionally, the BOLD platform was utilized for sponge DNA barcoding, focusing on species-level identification through a curated reference library of standardized mitochondrial COI (cytochrome oxidase I) genetic markers.

Phylogenetic analysis was conducted using the Unipro UGENE software platform (https://ugene.net/). DNA sequences obtained from GenBank and BOLD databases were together with the newly obtained sequences, were imported in FASTA format and aligned using the MAFFT (Multiple Sequence Alignment using Fast Fourier Transform) algorithm (Zhang et al., 2018). The Maximum Likelihood method was employed to construct the phylogenetic tree, incorporating bootstrap resampling with 1,000 replications. The resulting phylogenetic tree was visualized using FigTree v1.4.4, with posterior probabilities displayed at the nodes to indicate the support for the branches (Zou et al., 2024).

Crude Extracts Preparation for Antimicrobial Assays

The sponge samples were freeze-dried and ground into a fine powder using a mechanical grinder (Erngren et al., 2021). The powdered sponge material was subjected to solvent extraction using methanol, ethyl acetate, and dichloromethane to obtain bioactive compounds with varying polarities (Longo et al., 2025). The bioactive compounds recovery involved macerating the sponge powder in the respective solvents for 48 hours at room temperature with occasional agitation (Varijakzhan et al., 2021). The sponge extracts were then filtered using Whatman No. 1 filter paper (Sigma-Aldrich Company, St. Louis, Germany) and concentrated under reduced pressure using a rotary vacuum evaporator (BIOBASE Company, Jiangsu, China) at temperatures below 40°C to prevent thermal degradation of bioactive compounds (Mohd et al., 2023). The concentrated crude extracts were weighed and stored in sterile vials at 4°C until further analysis. The sponge extracts were assessed for their antimicrobial efficacy against test microorganisms: *Candida albicans* ATCC 10231, *Pseudomonas aeruginosa* ATCC 27853, *Escherichia coli* ATCC 25922, and

Staphylococcus aureus ATCC 25923. All microbial strains utilized in this study were procured from the Kenya Medical Research Institute (KEMRI), Kenya.

Assessment of *In Vitro* Antimicrobial Activity of Sponges' Crude Extracts

The antimicrobial screening of the sponge crude extracts against the test microorganisms was conducted using the Kirby-Bauer disk diffusion method (Latifah et al., 2021a). Sterile Mueller-Hinton agar (MHA) (HiMedia, Mumbai, India) was utilized as the medium for bacterial susceptibility testing (Salam et al., 2023). The MHA was prepared following the manufacturer’s specifications (38.0 g in 1000 mL of distilled water). For turbidity standardization, the bacterial test organisms were cultured in Mueller-Hinton broth (MHB) at 37°C for 18 hours with continuous agitation at 150 revolutions per minute (rpm) to ensure uniform bacterial suspension (Jalal et al., 2023). The MHB (HiMedia, Mumbai, India) was prepared according to the manufacturer’s guidelines (21 g in 1000 mL of purified water).

For *C. albicans,* Potato Dextrose Agar (PDA) (HiMedia, Mumbai, India) was used. *C. albicans* broth cultures were incubated at 30°C for 48 hours in Potato Dextrose Broth (PDB), prepared as per the manufacturer's protocol (24 g in 1000 mL of distilled water) for turbidity standardization. Microbial cultures were adjusted to a 0.5 McFarland turbidity standard, representing approximately 1.5×10^8 CFU/mL for bacterial cells and 1×10^6 CFU/mL for fungal cells, using sterile normal saline as the diluent. The normal saline was prepared by dissolving 9 g of NaCl in 1000 mL of deionized water.

MHA and PDA plates were inoculated with microbial test strains employing a sterile swab technique. Sponge crude extracts were solubilized in dimethyl sulfoxide (DMSO) to achieve working concentrations of 10 µL of 10 mg/mL sponge extracts in DMSO. Sterile paper discs (Oxoid, Thermo Scientific, United Kingdom) measuring 6 mm in diameter, were impregnated with 20 µL of sponge extracts and positioned on the inoculated agar plates (Manuel et al., 2021). Streptomycin (200 µg/ml) served as the positive control for bacterial strains, whereas fluconazole (10 mcg) was employed as the positive control for *C. albicans*, with DMSO functioning as the negative control (Domingos et al., 2024). Following incubation, plates were maintained at 37°C for bacterial strains and at 30°C for fungal strains, with measurements of inhibition zone diameters (IZD) conducted in millimeters using a digital caliper to evaluate antimicrobial efficacy (Gajic et al., 2022).

Determination of the minimum inhibitory concentration (MIC)

The broth microdilution method was employed to evaluate the antimicrobial efficacy of the marine sponge extracts against the selected pathogenic microbial strains (Anteneh et al., 2021). This method involved the preparation of a two-fold serial dilution of the marine sponge extracts in nutrient broth for antibacterial screening and in PDB for antifungal assessment (Balouiri et al., 2016). The test tubes were systematically labelled (Tube 2, Tube 3, Tube 4, Tube 5, Positive Control (+ve), and Negative Control (-ve). A stock solution of the sponge extract (Tube A) was initially prepared for subsequent dilutions. A volume of 2 mL of sterile broth was dispensed into each labelled test tube, beginning with the negative control. For the serial dilution, 2 mL of the sponge extract from Tube A was transferred into Tube 2 and mixed thoroughly. Subsequently, the process was repeated sequentially for Tubes 3 to 5. The concentration gradient of the stock solution

in the nutrient broth resulted in final concentrations of 0.625 mg mL^{-1}, 1.25 mg mL^{-1}, 2.5 mg mL^{-1}, 5 mg mL^{-1}, and 10 mg mL-1 of the sponge extracts (Júnior et al., 2021).

Following the preparation of the serial dilutions, 0.3 mL of the microbial suspension (*E. coli, S. aureus*, *P. aeruginosa,* and *C. albicans*), was introduced into all tubes except the negative control (Parvekar et al., 2020). The test tubes were then incubated under aerobic conditions at 37°C for 18 hours for bacterial strains and at 30°C for 48 hours for *C. albicans* to allow for microbial growth (Luo et al., 2021). After the incubation period, bacterial growth was assessed by measuring the optical density (OD) at 600 nm using a spectrophotometer (Shimadzu, Japan) (Mytilinaios et al., 2012). The minimum inhibitory concentration (MIC) was determined as the lowest concentration of the sponge extract that visibly inhibited bacterial growth (Kowalska and Dudek, 2021).

Evaluation of the minimum bactericidal concentration (MBC) and minimum fungicidal concentration (MFC)

The minimum bactericidal concentration (MBC) and minimum fungicidal concentration (MFC) were determined through the subculturing of wells from the MIC assays onto fresh agar plates (Abedini et al., 2020). Specifically, the bacterial cultures were incubated for 18 hours at 37 °C (Tuttle et al., 2021), while *C. albicans* cultures were incubated at 30 °C for 48 hours (Casagrande Pierantoni et al., 2021). Following incubation, the MHA plates were examined for the presence of surviving organisms. If the MBC of the sponge extracts was less than or equal to the MBC of the standard drug, the extract's activity was classified as either bactericidal or fungicidal (Giugliano et al., 2023).

Gas Chromatography-Mass Spectrometry (GC-MS) of Sponge Crude Extracts

The dried sponge extracts were reconstituted in analytical-grade n-hexane of high purity to attain a final concentration of 1 mg/mL (Rahman et al., 2024). Subsequently, the reconstituted samples were subjected to filtration through a 0.22 µm membrane filter to eliminate any particulate contaminants. A 1 µL aliquot of the prepared extract was injected into the GC-MS system for analytical assessment (Puah et al., 2019). The GC-MS system was equipped with an HP-5MS capillary column (5% phenyl methylpolysiloxane, 30 m × 0.25 mm i.d. × 0.25 µm film thickness) (Czarnowski et al., 2024). High-purity helium served as the carrier gas at a constant flow rate of 1 mL/min, and the analysis was performed in splitless mode to enhance sensitivity. The injector temperature was maintained at 250°C, while the oven temperature program commenced at an initial temperature of 50°C, held for 2 minutes, followed by a ramp rate of 10°C/min up to a final temperature of 300°C, where it was sustained for 5 minutes (Oliver, 2017).

The mass spectrometer was operated in Electron Ionization (EI) mode at an electron energy of 70 eV, with ion source temperature of 230°C and a quadrupole temperature of 150°C. The mass scan range was configured from 50 to 600 m/z, with a solvent delay period of 3 minutes. The mass spectra of the compounds detected in this study were systematically compared against established reference spectra derived from the National Institute of Standards and Technology (NIST) libraries (Valdez et al., 2021). An analysis of retention times and molecular ion peaks was conducted to authenticate the identities of the compounds. The relative abundance of bioactive compounds was quantified through peak area integration, enabling a precise determination of their prevalence. Identified compounds were categorized according to their chemical classes, including alkaloids, terpenoids, steroids, and fatty acids. The results were compared with previously reported marine-

derived bioactive compounds to assess their novelty and potential pharmaceutical applications (Mehbub et al., 2024).

Data analysis

A two-way analysis of variance (ANOVA) was performed to determine the presence of statistically significant differences in the diameters of inhibition zones across various marine sponge extracts. The data were recorded in triplicate and presented as mean ± standard deviation (SD) for the inhibition zones. Statistical tests of means sharing identical superscripts (*) within the column were considered not significantly different from one another, as determined through Fisher's Least Significant Difference (LSD) test at a 95% confidence level, following a post hoc analysis conducted using Minitab software version 21.4.1. Differences were classified as statistically significant at $P < 0.05$ ($\alpha = 0.05$).

The mass spectra of each compound analyzed by GC-MS were compared with the National Institute of Standards and Technology (NIST) spectral library for identification.

RESULTS

Morphology and taxonomy of marine sponges

These sponge samples were collected from Sii Island, Mundini, and Ras Kiromo study sites and taxonomically identified as *Biemna fistulosa, Callyspongia diffusa,* and *Haliclona fascigera*, respectively.

Upon removal from their natural habitat, the sponges exhibited significant morphological changes, including pronounced shrinkage and increased fragility. Additionally, a notable decline in their vibrant coloration was observed (Figure 1).

Figure 1: Photographic documentation of marine sponges from the Kenyan coastline, presented as follows: **(a)** *Biemna fistulosa* (Voucher specimen (BLSi 007) (in-situ) **(b)** *B. fistulosa* (detached) **(c)** *Callyspongia diffusa* (Voucher specimen BRMu 004) (in-situ) **(d)** *C. diffusa* (detached) **(e)** *Haliclona fascigera* (Voucher specimen BLUCh 014) (in-situ) **(f)** *Haliclona fascigera* (detached).

Biemna fistulosa sponge exhibited a robust, spongy and fibrous texture, with a growth pattern that encrusted and branched upwards (Figure 2). Initially, it presented a white-grey hue underwater, which upon exposure to air, turned black. The species, *B. ehrenbergi*, typically inhabited sandy shores and mangrove lagoons. Its structure was characterized by densely packed diactinal curved in styles (685.0-970.9-1235.7 x 7.9-19.0-32.8 μm). These spicules formed an irregular network, creating polygonal patterns. Additionally, microscleres such as C-shaped sigmas (30.4-41.0-50.3 μm), were also observed.

Figure 2: A. **Marine poriferan BLSi 007**, *Biemna fistulosa*; **B. sponge skeleton**: *(1sa):* perpendicular section; and *(1sb):* a tangential section (40x magnification); **C. sponge spicules:** *(1sc):* synapta plates; *(1sd)* and *(1se):* megascleres acanthostyles; *(1sf):* pentactines megascleres with digits at the tentacles; *(1sg):* curved oxeas; *(1sh):* curved styles; *(1si)* and *(1sk):* stauractines megascleres with digits at the tentacles; *(1sj):* pentactines megascleres with digits at the tentacles; *(1sl):* styles; *(1sm):* tabulated strongyles; *(1sn):* raphides; *(1so):* Microbiota (*Coscinodiscus radiatus*); *(1sp)* and *(1sr):* sterrasters; *(1sq):* microstrongyles; *(1ss):* strongyles; and *(1st):* dendroclones (unique to extinct sponge); **D. spongin fibers:** *(1fa):* simple elongated spongin fibers; *(1fb):* simple irregular spongin fibers; *(1fc):* Spongin fiber forming a fiber network on one end; *(1fd):* Spongin fiber with an irregular shape; *(1fe):* spongin fiber with a curved structure.

Figure 3: A. **Marine poriferan BRMu 004**, *Callyspongia diffusa*; **B. sponge skeleton**: *(2sa):* perpendicular section; and *(2sb):* a tangential section (40x magnification); **C. sponge spicules:** *(2sc)* and *(2sd):* anchorates; *(2se):* strongyles; *(2sf):* sterrasters; *(2sg):* acanthostyles; *(2sh):* curved oxeas; *(2si):* styles; *(2sj):* S sigmas; and *(2sk):* C sigmas; **D. spongin fibers:** *(2fa):* twisted thick spongin fibers; *(2fb):* Spongin fibers with bent thickened cell walls; *(2fc):* spongin fibers with a

complete bent (*Microcoleus vaginatus* attaching on the surface); *(2fd):* spongin fibers with an anastomosing system; and *(2fe):* spongin fibers with hard collagen material (spicules protruding).

Figure 4: A. Marine poriferan BLUCh 014, *Haliclona fascigera*; **B. sponge skeleton**: *(3sa):* perpendicular section; and *(3sb):* a tangential section (40x magnification); **C. sponge spicules:** *(3sc):* synapta plates; *(3sd)* and *(3sh):* stauractines megascleres with digits at the tentacles; *(3se);* styles; *(3sf):* tuberculated curved strongyles; *(3sg):* large plates of calcareous deposits; *(3si):* styles; **D. spongin fibers:** *(3fa):* spongin fibers with a thick flat structure; *(3fb):* branched spongin fibers; *(3fc):* twisted spongin fibers with an open transparent lumen; *(3fd):* spongin fibers with thickened cell walls and a smooth transparent lumen

Callyspongia diffusa also called the diffuse rope sponge exhibited a main skeleton composed of an isodictyl reticulation of collagenous spongin fibers, with diactinal spicules forming triangular meshes in the skeleton (Figure 3). The *C. diffusa* megascleres were represented by anchorates, acanthostyles (209.0-224.5-267.0 x 5.7-7.9-11.9 µm) and strongyles (163.9-178.2-179.3 x 4.9-7.4-10.7 µm), while the microscleres took the form of sterrasters. Additionally, the sponge was characterized by numerous raphides (149.3-168.6-279.8 µm).

The sponge *Haliclona fascigera* possessed a choanosomal skeleton, characterized by an isodictyal network of spicules intertwined with nodal spongin fibers, creating polygonal patterns (Figure 4). Its primary structural megascleres were exclusively oxeas (428-497.9-634.1 x 8.3-13.1-20.0 µm) and were embedded within spongin fibers. A significant number of raphides (114.2-163.0-179.3 µm), microstrongyles (128.0-148.0-178.4 µm) and synapta plates were observed. *H. fascigera* typically inhabited the shallow, sandy bottoms of lagoons and the vicinities of coral reefs.

Taxonomic Affiliation of the Marine Sponges

The genomic DNA extracted from marine sponge samples had NanoDrop quantifications ranging between 84.9 and 245.3 µg/mL. PCR amplification of the cytochrome c oxidase subunit I (COI) gene yielded amplicons within the expected size range of approximately 650 to 700 base pairs

(bp). All sponge specimens were classified within the phylum Porifera and identified as belonging to the genera *Callyspongia, Haliclona*, and *Biemna* based on percentage sequence similarities. A comparative analysis of the newly obtained mitochondrial Cytochrome Oxidase Subunit 1 (CO1) sequences (PQ329108, PQ997929, and PQ997931), conducted using BLASTn search against the GenBank database, showed sequence similarities of ≥99% with existing entries in the nucleotide sequence repository (Table 1). Each sponge sample formed a distinct sub-cluster (representing a specific genus), with a bootstrap support value of 100% (Table 1 and Figure 5). The marine sponge specimens BRMu 004 (PQ329108) from Mundini and BLUCh 014 (PQ997929) from Ras Kiromo were closely related to *Callyspongia diffusa* and *Haliclona fascigera*, respectively. These specimens had sequence identities of 100% and 99%, respectively (Table 1), and formed distinct subclusters, each supported by a bootstrap value of 100%, as depicted in the phylogenetic tree (Figure 5). The sponge specimen BLSi 007 (PQ997931) from Sii Island was closely related to the known sponge species *Biemna fistulosa*, with a sequence identity of 98%, and formed a subcluster with a bootstrap support value of 100% (Table 1 and Figure 5).

Table 1: Taxonomic affiliation of marine metazoan sponges with their closest phylogenetic relatives

Figure 5: Phylogenetic Relationships of Metazoan CO1 Sequences with Closely Related Sponge Species. The phylogenetic tree was rooted using *Stylissa carteri* (OX422287.1). Bootstrap values exceeding 50%, derived from 1000 replications, are indicated at the branch nodes. The scale bar represents 0.05 substitutions per nucleotide.

In Vitro **antibiotic and antifungal activity of the marine sponge crude extracts**

The marine sponge extracts were evaluated for their effectiveness against human pathogenic strains (*Candida albicans,* ATCC 10231, gram-negative bacteria *Pseudomonas aeruginosa* ATCC 25923 and *Escherichia coli* ATCC 25922 and the gram-positive bacterium *Staphylococcus aureus* ATCC 27853 (Tables 2, 3, and 4)). All the three crude organic extracts from the marine sponges (*B. fistulosa, C. diffusa* and *H. fascigera*) showed significant antimicrobial activity against at least

one of the four tested microorganisms, exceeding the efficacy of the positive control ($P < 0.05$) (Tables 2, 3 and 4). The methanolic extracts from the sponges *B. fistulosa* (28.00±3.5 mm) and *H. fascigera* (28.33±3.8 mm) exhibited a broad spectrum of antibacterial activity against *E. coli* that was higher than the positive control (27.67±0.9 mm) (Table 3). The inhibitory activity of ethyl acetate extracts *C. diffusa)* against *P. aeruginosa* was observed to be higher (29.33±2.4 mm) compared to that of the positive control (26.67±0.7 mm) (Table 4).

Methanolic extracts of *B. fistulosa* exhibited a higher (15.67±1.2mm) antifungal activity against *C. albicans* compared to the methanolic extract of *H. fascigera* (10.33±0.9 mm) and *C. diffusa* (9.67±2.2 mm) (Table 3). None of the dichloromethane and ethyl acetate extracts of the sponges exhibited antifungal activity against *C. albicans* (Table 2 and Table 4).

Table 2: Antimicrobial activities of dichloromethane organic crude extracts from the selected marine sponges against the tested human pathogens.

The marine sponges were coded based on their lifeform color and the study site of collection. The prefixes BL, BR, and BLU denoted black, brown, and blue sponges, respectively. These were followed by Si, Mu, and Ch, representing Sii Island, Mundini, and Ras Kiromo, respectively, along with the specimen number assigned to each sponge from the Kenyan coastline. Streptomycin was used as the positive control for bacterial strains, fluconazole as the positive control for *Candida albicans*, and Dimethyl Sulfoxide (DMSO) as the negative control. Data are expressed as means ± standard deviations (SD) from three replicates. Within each group, means sharing identical superscript letters indicate no significant difference at a 95% confidence level ($\alpha = 0.05$), as determined by Fisher's Least Significant Difference (LSD) test.

Table 3: Antimicrobial activities of methanolic organic crude extracts from the selected marine sponges against the tested human pathogens.

The tropical marine sponges were coded based on their lifeform color and collection site. The prefixes BL, BR, and BLU represented black, brown, and blue sponges, respectively, followed by Si, Mu, and Ch, indicating Sii Island, Mundini, and Ras Kiromo sampling sites. Each sponge was assigned a specimen number corresponding to its collection from the Kenyan coastline. Streptomycin served as the positive control for bacterial strains, fluconazole for *Candida albicans*, and Dimethyl Sulfoxide (DMSO) as the negative control. Data are presented as means ± standard deviations (SD) from three replicates. Within each group, means sharing the same superscript letter indicate no significant difference at a 95% confidence level (α = 0.05), as determined by Fisher's Least Significant Difference (LSD) test.

Table 4: Antimicrobial activities of ethyl acetate organic crude extracts from the selected marine sponges against the tested human pathogens.

The metazoan marine sponges were coded based on their lifeform color and collection site. The prefixes BL, BR, and BLU indicated black, brown, and blue sponges, respectively, followed by Si, Mu, and Ch, denoting Sii Island, Mundini, and Ras Kiromo sites. Each specimen was assigned a unique number corresponding to its collection from the Kenyan coastline. Streptomycin was employed as the positive control for bacterial strains, fluconazole for *Candida albicans*, and Dimethyl Sulfoxide (DMSO) as the negative control. Data are presented as means ± standard deviations (SD) from three replicates. Within each group, means with identical superscript letters signify no significant difference at a 95% confidence level (α = 0.05), as determined by Fisher's Least Significant Difference (LSD) test.

Evaluation of the sponge extracts for MIC, MBC and MFC

The Minimum Inhibitory Concentrations (MICs) of the sponge extracts were evaluated, demonstrating a range of values from 0.625 mg/ml to 10 mg/ml. Notably, streptomycin exhibited the lowest MIC values, with measurements of 1.94 mg/ml against *P. aeruginosa* and 1.36 mg/ml against *E. coli* (Table 5). The lowest MIC value (0.53±0.01 mg/ml) of methanolic extract of isolate *H. fascigera* was observed against *E. coli* compared to the standard drug/control (1.36±0.00 mg/ml) (Table 5). The MBC for all reference drugs was established at 2.5 mg/ml.

The organic crude extract obtained from *H. fascigera* demonstrated the most potent bactericidal activity, with a minimum bactericidal concentration (MBC) of 1.25 mg/mL, exceeding the efficacy of the standard drug streptomycin as well as the extracts derived from *C. diffusa* and *B. fistulosa*. Specifically, the MBC values of the sponge extracts were recorded as 2.5 mg/mL for *C. diffusa* and 5 mg/mL for *B. fistulosa*. In comparison, the MBC of the streptomycin reference drug was established at 2.5 mg/mL concentration. The methanolic extract of *H. fascigera* demonstrated the most potent fungicidal activity, with a minimum fungicidal concentration (MFC) of 2.5 mg/mL. Notably, the extracts from the both sponges *C. diffusa* and *B. fistulosa* exhibited an MFC of 10 mg/mL. The fluconazole reference drug showed an MFC comparable to that of *H. fascigera*, with a value of 2.5 mg/mL concentration.

Table 5: Minimum inhibitory concentrations (MIC) of dichloromethane, methanolic and ethyl acetate organic crude extracts of the selected marine sponges against the tested human pathogens.

The tropical marine sponges were coded based on their lifeform color and collection site. The prefixes BL, BR, and BLU represented black, brown, and blue sponges, respectively, followed by Si, Mu, and Ch, denoting Sii Island, Mundini, and Ras Kiromo sampling sites. Each specimen was assigned a unique identification number corresponding to its collection location along the Kenyan coastline. Streptomycin was utilized as the positive control for bacterial strains, fluconazole served

as the positive control for *Candida albicans*, and Dimethyl Sulfoxide (DMSO) functioned as the negative control. Data are presented as means ± standard deviations (SD) from three replicates. Within each group, means with identical superscript letters indicate no significant difference at a 95% confidence level ($\alpha = 0.05$), as determined by Fisher's Least Significant Difference (LSD) test.

GC-MS spectral analysis of the crude extract of the marine sponge extracts

The GC-MS analysis of the extracts from *B. fistulosa, C. diffusa,* and *H. fascigera* generated a spectral profile and chemical structures of the detected compounds (Table 6). The peak numbers in the chromatograms correspond to the identified compounds (Figures 6–8 and Table 7). The GC-MS chromatogram data identified a total of 114 compounds across the three sponge extracts (BLSi 007, BRMu 004, BLUCh 014). These compounds belong to 39 distinct chemical classes (Table 6)

The methanolic extract of the marine sponge *B. fistulosa* (BLSi 007) revealed a total of 47 chemical compounds from the GC-MS analysis (Figure 6). These compounds were grouped into alkane derivatives (2.1%), amino acid derivatives (6.4%), cyclic alkanes (2.1%), cyclic amines (2.1%), cyclic dipeptides (2.1%), cyclic esters (2.1%), diketones (2.1%), diazabicyclo compounds (2.1%), ergot alkaloids (4.3%), ester derivatives (6.3%), esterified fatty acids (10.6%), esterified forms of ascorbic acids (vitamin C) (2.1%), fatty acids (2.1%), heterocyclic amines (10.6%), heterocyclic compounds (10.6%), hydrazones (2.1%), imidazoles (2.1%), ketones (4.3%), long-chain saturated fatty acids (4.3%), monounsaturated fatty acids (10.6%), organic nitrogenous compounds (2.1%), pyrazole derivatives (2.1%), triazole derivatives (2.1%), and unsaturated fatty acids (2.1%).

The GC-MS chromatogram data identified a total of 62 chemical compounds from the ethyl acetate extract of the sponge *C. diffusa* (BRMu 004) (Figure 7). The detected compounds were classified into alkenes (1.6%), amino acid derivatives (9.5%), amines (4.8%), aromatic hydrocarbons (4.8%), carboxylic acid anhydrides (1.6%), chiral amino alcohols (1.6%), chiral organic compounds (1.6%), esters (12.7%), ergot alkaloids (1.6%), fatty alcohols (1.6%), heterocyclic organic compounds (27.0%), ketals (1.6%), organic esters (15.9%), phthalic acid esters (7.9%), polycyclic hydrocarbons (1.6%), quinolinedione derivatives (1.6%), triglycerides (1.6%), and α-ketoglutaric acids (1.6%).

The GC-MS analysis of the methanolic extract from the marine sponge *H. fascigera* (BLUCh 014) identified 37 chemical compounds (Figure 8). The chemical compound were categorized as acetylated amine alcohols (1.4%), amide compounds (2.7%), amide ester derivatives (13.5%), alkynes (2.7%), amino acid derivatives (2.7%), bicyclic chemical compounds (2.7%), cyclic alcohols (2.7%), cyclic alkanes (5.4%), cyclic amines (2.7%), epoxides (2.7%), esters (18.9%), heterocyclic amines (5.4%), heterocyclic boron-containing compounds (2.7%), heterocyclic compounds (10.8%), medium-chain fatty acids (2.7%), protected sugar derivatives (2.7%), pyrazole derivatives (2.7%), spiro compounds (2.7%), sugar alcohols (5.4%), synthetic compounds (2.7%), triazole derivatives (2.7%), and unsaturated carboxylic acids (2.7%).

Figure 6: GC-MS chromatogram analysis of the methanolic extract of *Biemna fistulosa* (BLSi 007), highlighting four potent bioactive compounds

Figure 7: GC-MS chromatogram analysis of the ethyl acetate extract of *Callyspongia diffusa* (BRMu 004), identifying six secondary bioactive compounds.

Figure 8: GC-MS chromatogram analysis of the methanolic extract of *Haliclona fascigera* (BLUCh 014), revealing five bioactive compounds.

In this study, GC-MS chromatogram analysis of the methanolic extract from *Biemna fistulosa* (BLSi 007) identified four potent bioactive compounds: (2S,6R)-2,6-dibutyl-4-methylpiperidine, pyrrolo [1,2-a] pyrazine-1,4-dione, hexahydro-3-(phenylmethyl), 1-(2-ethyl-1,2,4-triazol-3-yl) ethanamine, and 3,6-diisopropylpiperazin-2,5-dione (Figure 6 and Table 7).

Similarly, GC-MS analysis of the ethyl acetate extract from *Callyspongia diffusa* (BRMu 004) revealed six secondary bioactive compounds, including 7-n-pentadecylaminomethyl-6-hydroxy-5,8-quinolinedione, eicosanoic acid butyl ester, pyrrolo [1,2-a] pyrazine-1,4-dione, hexahydro-3-(phenylmethyl), 4H-pyran-4-one, 2,2'-isopropylidenebis [3-methoxy-6-methyl], (2S,6R)-2,6-dibutyl-4-methylpiperidine, and phthalic acid, 2-ethylhexyl tetradecyl ester (Figure 7 and Table 7).

The methanolic extract from *Haliclona fascigera* (BLUCh 014) exhibited five bioactive compounds: xylitol, 1-(2-ethyl-1,2,4-triazol-3-yl) ethanamine, pyrrolo [1,2-a] pyrazine-1,4-dione, hexahydro-3-(phenylmethyl), (2S,6R)-2,6-dibutyl-4-methylpiperidine, and n-propyl 9-tetradecenoate (Figure 8 and Table 6). Notably, the bioactive compounds (2S,6R)-2,6-dibutyl-4-methylpiperidine and pyrrolo [1,2-a] pyrazine-1,4-dione, hexahydro-3-(phenylmethyl) were consistently present in all three sponge extracts.

Table 6: Classification of marine sponge compounds identified via GC-MS in organic extracts from *Biemna fistulosa, Callyspongia diffusa,* and *Haliclona fascigera* collected from Kenyan waters.

Table 7: Characteristics and antimicrobial activity of selected sponges' natural products identified in the GC-MS analysis of *Biemna fistulosa, Callyspongia diffusa,* and *Haliclona fascigera* organic extracts.

DISCUSSION

In this study, the morphological identification of *Biemna fistulosa*, *Callyspongia diffusa*, and *Haliclona fascigera* revealed distinct skeletal structures and spicule compositions, aiding in their taxonomic classification. *B. fistulosa* exhibited a fibrous, encrusting growth pattern with curved diactinal styles and C-shaped sigma microscleres, while *C. diffusa* featured a spongin fiber

reticulation with triangular spicule meshes, and *H. fascigera* is characterized by an isodictyal network of oxeas, raphides, and microstrongyles, commonly found in sandy lagoons and coral reef environments. A similar study used spicular analysis to investigate the sponge spicule assemblage, including *Biemna, Callyspongia*, and *Haliclona* species, in the lagoon reef of Bocas del Toro, Panama (Łukowiak, 2016). In Simeulue Island, Aceh Province, Indonesia conducted a morphological study was conducted on over twenty species of marine sponges. Their findings included notable species such as *Carteriospongia foliascens, Biemna fortis, Paratetilla aruensis, Oceanapia sp., Petrosia sp., Haliclona oculata,* and *Haliclona fascigera* (Setiawan et al., 201[illegible]

In addition, this study used a DNA barcoding, technique and identified three genera (*Biemna, Callyspongia*, and *Haliclona*) within the class Demospongiae. The complementary approaches significantly improved the precision of taxonomic classification for the marine sponges. The phylogenetic analysis clustered the sponge samples into three sub-clusters, with each representing a distinct genus. The specimen *Callyspongia* sp. BRMu004 (PQ329108) formed a distinct sub-cluster (supported by 100% bootstrap value) with members of the genus *Callyspongia* and had 100% sequence identity with *Callyspongia diffusa* (KX454494) (Figure 5; Table 1). On the other hand, *Haliclona* sp. BLUCh014 (PQ997929) clustered together with members of the genus *Haliclona* that had 99% sequence similarity. The specimen *Biemna* sp. BLSi007 (PQ997931) formed its sub-cluster (supported by 100% bootstrap value) with species from the genus *Biemna.* The formation of separate sub-clusters by the three sponge specimens on the phylogenetic tree indicated that they were representing distinct sponge species. A previous study examined species of shallow Hawaiian sponge fauna in the United States and identified *Biemna fistulosa* and *Callyspongia diffusa* using DNA barcoding (Núñez et al., 2017)**.**

The GC-MS chromatogram data indicatted a total of 114 marine sponge chemical compounds from genera *Biemna, Callyspongia* and *Haliclona*. Notably, this study confirmed that 11.4% of the identified compounds had previously demonstrated antifungal, antibacterial, and antiviral bioactivity. A previous study demonstrated that *Callyspongia* species from the Red Sea exhibited antimicrobial properties, with bioactivity observed in the methanolic extract, its various fractions, and particularly in extracts purified from the dichloromethane fraction (Musa et al., 2022). Marine sponges belonging to the genus *Haliclona* are recognized for their capacity to biosynthesize a wide spectrum of secondary bioactive compounds such as sesquiterpenoid quinols, sterols, glycosphingolipids, and bioactive alkaloid compounds (Varijakzhan et al., 2021).

The findings in this study indicate that the ethyl acetate extracts of *C. diffusa* exhibited notable inhibitory activity against *P. aeruginosa*. The GC-MS analysis identified 62 chemical compounds in the ethyl acetate extract of *C. diffusa*, of which 9.7% have been previously reported to exhibit antimicrobial activity. These bioactive compounds include pyrrolo [1,2-a] pyrazine-1,4-dione, hexahydro-3-(phenylmethyl) (Al-Askar et al., 2024 and Kiran et al., 2018), (2S,6R)-2,6-Dibutyl-4-methylpiperidine (Ramya and Sethumadhavan, 2022), 7-n-Pentadecylaminomethyl-6-hydroxy-5,8-quinolinedione (Kadela et al., 2019), 4H-Pyran-4-one, 2,2'-isopropylidenebis [3-methoxy-6-methyl] (Almalki, 2023), phthalic acid, 2-ethylhexyl tetradecyl ester (Huang et al., 2021) and pyrrolo [1,2-a] pyrazine-1,4-dione; hexahydro-3-(2-methylpropyl)) (Askar et al., 2024 and Kiran et al., 2018). A similar study identified 212 bioactive compounds from the genus *Callyspongia,* and 109 molecules were reported to exhibit bioactivity (Sousa et al., 2021).

Notably, the methanolic extracts from the marine sponges *B. fistulosa* and *H. fascigera* show significant antibacterial activity against *E. coli.* Among the compounds identified in this study, 4.4% exhibited antibacterial activity, including pyrrolo[1,2-a] pyrazine-1,4-dione, hexahydro-3-(phenylmethyl) (Askar et al., 2024 and Kiran et al., 2018), 1-(2-ethyl-1,2,4-triazol-3-yl) ethanamine (Gupta et al., 2023), 7-n-pentadecylaminomethyl-6-hydroxy-5,8-quinolinedione (Kadela et al., 2019), 4H-pyran-4-one, 2,2'-isopropylidenebis [3-methoxy-6-methyl] (Almalki, 2023), and xylitol (Gasmi et al., 2020 and Salli et al., 2019). In a related study, extracts from *H. fascigera* sourced in Indonesia proved effective in inhibiting the growth of *S. aureus* and *E. coli* (Sugrani et al., 2019). Another study also revealed the antibacterial activities of crude extracts of *H. fascigera* against three shrimp pathogenic bacteria (Latifah et al., 2021). Elsewhere, Soulange et al. (2014)

revealed that the organic sponge extracts exhibited greater antibacterial activity than the standard antibiotic against *S. aureus* and *E. coli.*

Candida albicans is the predominant causative agent of candidiasis, accounting for approximately 70% of fungal infections worldwide and contributing to the annual mortality of over 1.6 million people due to fungal diseases (Richardson, 2022). On one hand, *B. fistulosa* demonstrated more potent bioactive compounds capable of inhibiting *C. albicans* growth compared to *C. diffusa* and *H. fascigera*. Notably, 4.4% of the identified bioactive compounds were previously reported to possess antifungal properties, including pyrrolo [1,2-a] pyrazine-1,4-dione, hexahydro-3-(phenylmethyl) (Askar et al., 2024 and Kiran et al., 2018), pyrrolo[1,2-a] pyrazine-1,4-dione; hexahydro-3-(2-methylpropyl) (Al-Askar et al., 2024 and Kiran et al., 2018), 1-(2-ethyl-1,2,4-triazol-3-yl) ethanamine (Gupta et al., 2023), 7-n-pentadecylaminomethyl-6-hydroxy-5,8-

quinolinedione (Kadela et al., 2019), and n-propyl 9-tetradecenoate (Kadhim et al., 2016). Xestodecalactones B compounds isolated from Xestospongia *exigua* in the Bali Sea demonstrated antifungal activity against *C. albicans* (Varijakzhan et al., 2021). Similarly, nortetillapyrone, a tetrahydrofurylhydroxypyran-2-one derived from *Haliclona cymaeformis*, exhibited antifungal efficacy against various fungal pathogens with distinct MIC values (Ngece et al., 2024). Furthermore, polyketide compounds such as woodylides A and C from *Plakortis simplex*, as well as theonellamide G, swinholide I, and hurghadolide A from *Theonella swinhoei*, along with tetramic acid glycosides aurantosides G and I, demonstrated significant antifungal activity, underscoring the potential of marine sponges as reservoirs of antifungal compounds (Yu et al., 2012).

The decanoic acid, 10-(2-hexylcyclopropyl), a fatty acid obtained from the methanolic extract of *B. fistulosa*, has been used in identifying bacteria and in research for studying its role in bacterial cell membrane protection and metabolism (Casillas et al., 2021). Furthermore, 9-octadecenoic acid, 1,2,3-propanetriyl ester E, E, E, a triolein compound, was extracted from the ethyl acetate extract of *C. diffusa*. This compound holds pharmaceutical significance as a carrier or excipient in drug formulations and is also employed as an emollient and moisturizing agent in dermatological products (Kumar and Sharma, 2023). In a previous study, the bioactive compound 9-Octadecenoic acid, 1,2,3-propanetriyl ester (E, E, E) was discovered in *Cassia angustifolia* and demonstrated significant antimicrobial activity (Marzoqi et al., 2016).

In our study, the methanolic extract of *H. fascigera* exhibited a lower minimum inhibitory concentration (MIC), categorizing it as a potent inhibitor with enhanced efficacy in suppressing

the growth of *Escherichia coli* compared to the standard drug. Moreover, the extract demonstrated significant bactericidal activity, with a minimum bactericidal concentration (MBC) of 1.25 mg/mL, which was substantially lower than the MBC values recorded for *C. diffusa* and *B. fistulosa*. Additionally, this research established that the ethyl acetate extract derived from *C. diffusa* demonstrated bactericidal activity against *Pseudomonas aeruginosa*, with an MBC value of 2.5 mg/mL. Marine sponge extracts from a study of the waters of Mauritius exhibited low MBC values, indicating that these extracts could serve as a potential approach to traditional bacterial infection management strategies (Beesoo et al., 2017). In a similar study, *H. fascigera*, collected from Bidong Island, Malaysia, showed positive results in all in vitro biological studies and exhibited higher antibacterial activities compared to other sponges in the study (Jamaludin et al., 2023). The research also revealed that the butanol fraction of *Biemna* sp. exhibited significant antibacterial activity, with an MIC value of 0.091 mg/ml against *Escherichia coli* (Soulange et al., 2014).

The findings of this study indicate that the methanolic extract of *H. fascigera* exhibited the most potent fungicidal activity, with a minimum fungicidal concentration (MFC) of 2.5 mg/mL. This suggests that the extracts of *H. fascigera* are more effective at eliminating *C. albicans* at lower concentrations compared to the other extracts evaluated. Furthermore, research conducted in Biak, Indonesia, demonstrated that the ethyl acetate extracts of *Fascaplysinopsis* sp. and *Haliclona* sp. possess notable antifungal activity against *C. albicans* (Kasitowati et al., 2019). Additionally, investigations on marine sponges from the Ratnagiri coast of India reported moderate antifungal activity against *C. albicans* (Kumar, 2022).

CONCLUSION AND RECOMMENDATIONS

This research study underscores the significance of marine sponges as a promising reservoir of antimicrobial agents. Extracts derived from *B. fistulosa*, *C. diffusa*, and *H. fascigera* exhibited pronounced antibacterial activity compared to the positive control. Furthermore, the methanolic extract of *H. fascigera* demonstrated the most potent antifungal activity among the marine sponge extracts evaluated. The findings of this study suggest that marine sponges from Kenyan waters possess notable therapeutic potential, presenting valuable lead compounds for drug discovery and development. More studies should focus on the mechanisms of action and toxicity of pure leads isolated from sponges at the molecular level, which is important to give direction for lead improvement and further drug development.

CONFLICT OF INTERESTS

The authors have not declared any conflict of interest.

AUTHORS' CONTRIBUTION

Conceptualization, T.N.W., H.M.M., J.K.N, C.M.A, and C.M.K; writing— original draft preparation, T.N.W.; writing—review and editing, T.N.W., H.M.M., J.K.N, C.M.A and C.M.K. All authors have read and agreed to publish the manuscript.

FUNDING

We sincerely thank the Kenya Marine and Fisheries Research Institute (KMFRI) for their invaluable support (GOK-PC Target C82 39-1).

DATA AVAILABILITY STATEMENT

The data presented in this study are available upon request from the corresponding authors.

ACKNOWLEDGEMENTS

We are profoundly grateful to the Kenya Marine and Fisheries Research Institute (KMFRI) and the Technical University of Mombasa (TUM) for their unwavering support. Their outstanding laboratory facilities were instrumental in the successful completion of this research, enabling us to achieve our objectives effectively.

REFERENCES

Abdelkhalek A, Aseel DG, Király L, Künstler A, Moawad H, Al-Askar AA. Induction of systemic resistance to Tobacco mosaic virus in tomato through foliar application of Bacillus amyloliquefaciens strain TBorg1 culture filtrate. Viruses. 2022;14(8). doi: 10.3390/v14081830.

Abedini A, Colin M, Hubert J, Charpentier E, Angelis A, Bounasri H, Bertaux B, Kotland A, Reffuveille F, Nuzillard JM, Renault JH, et al. Abundant extractable metabolites from temperate tree barks: The specific antimicrobial activity of Prunus avium extracts. Antibiotics. 2020;9(3). doi: 10.3390/antibiotics9030111.

Ahuja V, Macho M, Ewe D, Singh M, Saha S, Saurav K. Biological and pharmacological potential of xylitol: A molecular insight of unique metabolism. Foods. 2020;9(11). doi: 10.3390/foods9111592.

Al-Askar AA, Al-Otibi FO, Abo-Zaid GA, Abdelkhalek A. Pyrrolo[1,2-a]pyrazine-1,4-dione, hexahydro-3-(2-methylpropyl), as the primary secondary metabolite of Bacillus spp., could be an effective antifungal agent against the soil-borne fungus Sclerotium bataticola. Egypt J Chem. 2024;67(13):1009–1022. doi: 10.21608/ejchem.2024.325664.10571.

Al-Marzoqi AH, Hadi MY, Hameed IH. Determination of metabolites products by Cassia angustifolia and evaluation of antimicrobial activity. J Pharmacogn Phytother. 2016;8(2). doi: 10.5897/JPP2015.0367.

Al-Shuhaib MBS, Hashim HO. Mastering DNA chromatogram analysis in Sanger sequencing for reliable clinical analysis. J Genet Eng Biotechnol. 2023;21(1). doi: 10.1186/s43141-023-00587-6.

Anteneh YS, Yang Q, Brown MH, Franco CMM. Antimicrobial activities of marine sponge-associated bacteria. Microorganisms. 2021;9(1):1–19. doi: 10.3390/microorganisms9010171.

Ayon NJ. High-throughput screening of natural product and synthetic molecule libraries for antibacterial drug discovery. Metabolites. 2023;13(5). doi: 10.3390/metabo13050625.

Bach TMH, Takagi H. Properties, metabolisms, and applications of L-proline analogues. Appl Microbiol Biotechnol. 2013;97(15). doi: 10.1007/s00253-013-5022-7.

Balouiri M, Sadiki M, Ibnsouda SK. Methods for in vitro evaluating antimicrobial activity: A review. J Pharm Anal. 2016;6(2):71–79. doi: 10.1016/j.jpha.2015.11.005.

Beesoo R, Bhagooli R, Neergheen-Bhujun VS, Li WW, Kagansky A, Bahorun T. Antibacterial and antibiotic potentiating activities of tropical marine sponge extracts. Comp Biochem Physiol C Toxicol Pharmacol. 2017;196. doi: 10.1016/j.cbpc.2017.04.001.

Cai W, MacDonald B, Korabik M, Gradin I, Neave EF, Harper LR, et al. Biofouling sponges as natural eDNA samplers for marine vertebrate biodiversity monitoring. Sci Total Environ. 2024;946. doi: 10.1016/j.scitotenv.2024.174148.

Carrier TJ, Maldonado M, Schmittmann L, Pita L, Bosch TCG, Hentschel U. Symbiont transmission in marine sponges: reproduction, development, and metamorphosis. BMC Biol. 2022;20(1):100. doi: 10.1186/s12915-022-01291-6.

Casagrande Pierantoni D, Corte L, Casadevall A, Robert V, Cardinali G, Tascini C. How does temperature trigger biofilm adhesion and growth in Candida albicans and two non-Candida albicans Candida species? Mycoses. 2021;64(11). doi: 10.1111/myc.13291.

Casillas-Vargas G, Ocasio-Malavé C, Medina S, Morales-Guzmán C, Del Valle RG, Carballeira NM, Sanabria-Ríos DJ. Antibacterial fatty acids: An update of possible mechanisms of action and implications in the development of the next-generation of antibacterial agents. Prog Lipid Res. 2021;82. doi: 10.1016/j.plipres.2021.101093.

Christian OE, Perry DA, Telchy AI, Walton PN, Williams D. Bioactive compounds isolated from a marine sponge selectively inhibit Neisseria gonorrhoeae. Antibiotics. 2024;13(12). doi: 10.3390/antibiotics13121229.

Cleary DFR, de Voogd NJ, Stuij TM, Swierts T, Oliveira V, Polónia ARM, et al. A study of sponge symbionts from different light habitats. Microb Ecol. 2023;86(4). doi: 10.1007/s00248-023-02267-x.

Costello MJ, Bouchet P, Boxshall G, Fauchald K, Gordon D, Hoeksema BW, et al. Global coordination and standardisation in marine biodiversity through the World Register of Marine Species (WoRMS) and related databases. PLoS One. 2013;8(1). doi: 10.1371/journal.pone.0051629.

Czarnowski P, Mikula M, Ostrowski J, Żeber-Lubecka N. Gas chromatography–mass spectrometry-based analyses of fecal short-chain fatty acids (SCFAs): A summary review and own experience. Biomedicines. 2024;12(8). doi: 10.3390/biomedicines12081904.

Daniels BN, Nurge J, Sleeper O, Lee A, López C, Christie MR, et al. Genomic DNA extraction optimization and validation for genome sequencing using the marine gastropod Kellet's whelk. PeerJ. 2023;11. doi: 10.7717/peerj.16510.

de Sousa LHN, de Araújo RD, Sousa-Fontoura D, Menezes FG, Araújo RM. Metabolites from marine sponges of the genus Callyspongia: Occurrence, biological activity, and NMR data. Mar Drugs. 2021;19(12). doi: 10.3390/md19120663.

Domingos LTS, de Moraes DC, Santos MFC, Curvelo JAR, Bayona-Pacheco B, Marquez EA, et al. Batzelladine D, a marine natural product, reverses the fluconazole resistance phenotype mediated by transmembrane transporters in Candida albicans and interferes with its biofilm: An in vitro and in silico study. Mar Drugs. 2024;22(11). doi: 10.3390/md22110502.

Erngren I, Smit E, Pettersson C, Cárdenas P, Hedeland M. The effects of sampling and storage conditions on the metabolite profile of the marine sponge Geodia barretti. Front Chem. 2021;9. doi: 10.3389/fchem.2021.662659.

Esposito R, Federico S, Bertolino M, Zupo V, Costantini M. Marine Demospongiae: A challenging treasure of bioactive compounds. Mar Drugs. 2022;20(4). doi: 10.3390/md20040244.

Folkers M, Rombouts T. Sponges revealed: A synthesis of their overlooked ecological functions within aquatic ecosystems. In: Jungblut S, Liebich V, Bode-Dalby M, editors. YOUMARES 9 - The Oceans: Our Research, Our Future. Springer International Publishing; 2020. p. 181–193. doi: 10.1007/978-3-030-20389-4_9.

Gajic I, Kabic J, Kekic D, Jovicevic M, Milenkovic M, Mitic Culafic D, et al. Antimicrobial susceptibility testing: A comprehensive review of currently used methods. Antibiotics. 2022;11(4). doi: 10.3390/antibiotics11040427.

García-Vilas JA, Martínez-Poveda B, Quesada AR, Medina MA. Aeroplysinin-1, a sponge-derived multi-targeted bioactive marine drug. Mar Drugs. 2016;14(1). doi: 10.3390/md14010001.

Garritano AN, Majzoub ME, Ribeiro B, Damasceno T, Modolon F, Messias C, et al. Species-specific relationships between deep sea sponges and their symbiotic Nitrosopumilaceae. ISME J. 2023;17(9). doi: 10.1038/s41396-023-01439-4.

Gasmi Benahmed A, Gasmi A, Arshad M, Shanaida M, Lysiuk R, Peana M, et al. Health benefits of xylitol. Appl Microbiol Biotechnol. 2020;104(17). doi: 10.1007/s00253-020-10708-7.

Giugliano R, Della Sala G, Buonocore C, Zannella C, Tedesco P, Palma Esposito F, et al. New imidazolium alkaloids with broad spectrum of action from the marine bacterium Shewanella aquimarina. Pharmaceutics. 2023;15(8). doi: 10.3390/pharmaceutics15082139.

Goldschmidt P, Degorge S, Merabet L, Chaumeil C. Enzymatic treatment of specimens before DNA extraction directly influences molecular detection of infectious agents. PLoS One. 2014;9(6). doi: 10.1371/journal.pone.0094886.

Govinden-Soulange J, Marie D, Kauroo S, Beesoo R, Ramanjooloo A. Antibacterial properties of marine sponges from Mauritius waters. Trop J Pharm Res. 2014;13(2):249–254. doi: 10.4314/tjpr.v13i2.13.

Gupta O, Pradhan T, Chawla G. An updated review on diverse range of biological activities of 1,2,4-triazole derivatives: Insight into structure activity relationship. J Mol Struct. 2023;1274. doi: 10.1016/j.molstruc.2022.134487.

Hadi TA, Suharsono -, Hadiyanto -, Budiyanto A, Wentao N. The morphological and species diversity of sponges on coral reef ecosystems in the Lembeh Strait, Bitung. Mar Res Indones. 2016;40(2). doi: 10.14203/mri.v40i2.45.

Hamouda RA, Eshmawi BA, Saddiq AA. Anticancer, antioxidant, and antimicrobial activities of nanoemulsions based on water-in-olive oil and loaded on biogenic silver nanoparticles. Green Process Synth. 2024;13(1). doi: 10.1515/gps-2024-0026.

Handayani D, Aminah I, Pontana Putra P, Eka Putra A, Arbain D, Satriawan H, et al. The depsidones from marine sponge-derived fungus Aspergillus unguis IB151 as an anti-MRSA agent: Molecular docking, pharmacokinetics analysis, and molecular dynamic simulation studies. Saudi Pharm J. 2023;31(9). doi: 10.1016/j.jsps.2023.101744.

Handayani D, Sandrawaty N, Murniati M, Regina R. Screening of endophytic bacteria isolated from marine sponge Haliclona fascigera for inhibition against clinical isolates of methicillin-resistant Staphylococcus aureus (MRSA). J Appl Pharm Sci. 2015;5(9). doi: 10.7324/JAPS.2015.50926.

Holub M, Birnie A, Japaridze A, van der Torre J, den Ridder M, de Ram C, et al. Extracting and characterizing protein-free megabase-pair DNA for in vitro experiments. Cell Rep Methods. 2022;2(12). doi: 10.1016/j.crmeth.2022.100366.

Hong LL, Ding YF, Zhang W, Lin HW. Chemical and biological diversity of new natural products from marine sponges: a review (2009–2018). Mar Life Sci Technol. 2022;4(3):356–372. doi: 10.1007/s42995-022-00132-3.

Huang L, Zhu X, Zhou S, Cheng Z, Shi K, Zhang C, Shao H. Phthalic acid esters: Natural sources and biological activities. Toxins. 2021;13(7). doi: 10.3390/toxins13070495.

Huigens RW. The development of ageliferin inspired small molecules as antibiofilm and antibacterial agents against multidrug-resistant bacterial pathogens. 2009. doi: 10.87760395.

Jalal N, Hariri S, Abdel-razik N, Alzahrani A, Khan S, Bantun F. Synergistic effect of biogenic silver nanoparticles and antibiotics against multidrug-resistant Pseudomonas aeruginosa. Egypt Acad J Biol Sci C Physiol Mol Biol. 2023;15(1):319–337. doi: 10.21608/eajbsc.2023.303331.

Jamaludin NA, Bakar K, Saidin J. In vitro biological activity of three marine sponges from Theonella and Haliclona genera collected from Bidong Island, Terengganu, Malaysia. Malays Appl Biol. 2023;52(2). doi: 10.55230/mabjournal.v52i2.2559.

Jensen M, Keding R, Yue Y. Microscopic features of biologically formed amorphous silica. On Biomimetics. 2011. doi: 10.5772/24951.

Jia J, Yao J, Kong J, Yu A, Wei J, Dong Y, et al. 2,5-Diketopiperazines: A review of source, synthesis, bioactivity, structure, and MS fragmentation. Curr Med Chem. 2022;30(9). doi: 10.2174/0929867329666220801143650.

Júnior ACV, de Castro Nogueira Diniz Pontes M, Barbosa JP, Höfling JF, Araújo RM, Boniek D, et al. Antibiofilm and anti-Candidal activities of the extract of the marine sponge Agelas dispar. Mycopathologia. 2021;186(6). doi: 10.1007/s11046-021-00591-9.

Kadela-Tomanek M, Bębenek E, Chrobak E, Boryczka S. 5,8-Quinolinedione scaffold as a promising moiety of bioactive agents. Molecules. 2019;24(22). doi: 10.3390/molecules24224115.

Kadhim MJ, Mohammad GJ, Mohammed GJ, Hussein H. Analysis of bioactive metabolites from Candida albicans using (GC-MS) and evaluation of antibacterial activity. Int J Pharm Clin Res. 2016;8(7). Available from: www.ijpcr.com..

Kanakaraj I, Jewell DL, Murphy JC, Fox GE, Willson RC. Removal of PCR error products and unincorporated primers by metal-chelate affinity chromatography. PLoS One. 2011;6(1). doi: 10.1371/journal.pone.0014512.

Kasitowati RD, Wittriansyah K, Trianto A, Pratiwi DC, Panjaitan MAP. Antifungal activity of marine sponges (Class Demospongiae) collected from Biak, Indonesia. IOP Conf Ser Earth Environ Sci. 2019;236(1). doi: 10.1088/1755-1315/236/1/012102.

Kiran GS, Priyadharsini S, Sajayan A, Ravindran A, Selvin J. An antibiotic agent pyrrolo[1,2-a]pyrazine-1,4-dione, hexahydro isolated from a marine bacterium Bacillus tequilensis MSI45 effectively controls multi-drug resistant Staphylococcus aureus. RSC Adv. 2018;8(32). doi: 10.1039/c8ra00820e.

Kowalska-Krochmal B, Dudek-Wicher R. The minimum inhibitory concentration of antibiotics: Methods, interpretation, clinical relevance. Pathogens. 2021;10(2):1–21. doi: 10.3390/pathogens10020165.

Kumar MS. Antifungal and antibacterial activity of marine sponges from Ratnagiri coast of India. J Med Mycol. 2022;32(4). doi: 10.1016/j.mycmed.2022.101305.

Kumar N, Sharma S. Pharmacology, ethnopharmacology, and phytochemistry of medicinally active Moringa oleifera: A review. Nat Prod J. 2023;13(8). doi: 10.2174/2210315513666230301094259.

Kumar S, Chandra P, Bajpai V, Singh A, Srivastava M, Mishra DK, Kumar B. Rapid qualitative and quantitative analysis of bioactive compounds from Phyllanthus amarus using LC/MS/MS techniques. Ind Crops Prod. 2015;69. doi: 10.1016/j.indcrop.2015.02.012.

Kurian A, Elumalai P. Marine sponge: Natural reservoir of a myriad of bioactive compounds. Int J Pharm Pharm Res. 2017;8(2).

Latifah LA, Tahir A, Soekamto NH. Antibacterial assay of crude extracts from marine sponge Haliclona fascigera in Badi Island of Spermonde Archipelago against shrimp pathogenic bacteria. IOP Conf Ser Earth Environ Sci. 2021;763(1). doi: 10.1088/1755-1315/763/1/012029.

Latifah LA, Tahir A, Soekamto NH. Antibacterial assay of crude extracts from marine sponge Haliclona fascigera in Badi Island of Spermonde Archipelago against shrimp pathogenic bacteria. IOP Conf Ser Earth Environ Sci. 2021;763(1). doi: 10.1088/1755-1315/763/1/012029.

Longo C, Pierri C, Trani R, Mercurio M, Nonnis Marzano C, Corriero G, et al. Toward a green strategy of sponge mariculture and bioactive compounds recovery. Sci Rep. 2025;15(1):5999. doi: 10.1038/s41598-025-90192-z.

Łukowiak M. Spicular analysis of surficial sediments as a supplementary tool for studies of modern sponge communities. Helgol Mar Res. 2016;70(1). doi: 10.1186/s10152-016-0459-6.

Luo Y, McAuley DF, Fulton CR, Pessoa JS, McMullan R, Lundy FT. Targeting Candida albicans in dual-species biofilms with antifungal treatment reduces Staphylococcus aureus and MRSA in vitro. PLoS One. 2021;16(4). doi: 10.1371/journal.pone.0249547.

Maldonado M, Ribes M, van Duyl FC. Nutrient fluxes through sponges: Biology, budgets, and ecological implications. Adv Mar Biol. 2012;62. doi: 10.1016/B978-0-12-394283-8.00003-5.

Manuel RP, Tania GG, Rafael SP, Antonio PE, José BM, Asteria LÁ, et al. In vitro development of a new sponge-based delivery system for intracanal antimicrobial administration in endodontic treatment. J Clin Med. 2021;10(12). doi: 10.3390/jcm10122725.

Marín DV, Castillo DK, López-Lavalle LAB, Chalarca JR, Pérez CR. An optimized high-quality DNA isolation protocol for Spodoptera frugiperda J.E. Smith (Lepidoptera: Noctuidae). MethodsX. 2021;8. doi: 10.1016/j.mex.2021.101255.

Mehbub MF, Yang Q, Cheng Y, Franco CMM, Zhang W. Marine sponge-derived natural products: Trends and opportunities for the decade of 2011-2020. Front Mar Sci. 2024;11. doi: 10.3389/fmars.2024.1462825.

Moghaddam JA, Dávila-Céspedes A, Kehraus S, Crüsemann M, Köse M, Müller CE, König GM. Cyclopropane-containing fatty acids from the marine bacterium Labrenzia sp. 011 with antimicrobial and GPR84 activity. Mar Drugs. 2018;16(10). doi: 10.3390/md16100369.

Mohd Ramzi M, Rahman NIA, Rawi NN, Bhubalan K, Ariffin F, Mazlan NW, et al. Antifouling potential of Diadema setosum and Sonneratia lanceolata extracts for marine applications. J Mar Sci Eng. 2023;11(3). doi: 10.3390/jmse11030602.

Musa A, Abdelgawad MA, Shaker ME, El-Ghorab AH, Parambi DGT, Hamed AA, et al. Screening and molecular docking of bioactive metabolites of the Red Sea sponge Callyspongia siphonella as potential antimicrobial agents. Antibiotics. 2022;11(12). doi: 10.3390/antibiotics11121682.

Musembi P, Fulanda B, Kairo J, Githaiga M. Species composition, abundance, and fishing methods of small-scale fisheries in the seagrass meadows of Gazi Bay, Kenya. J Indian Ocean Reg. 2019;15(2). doi: 10.1080/19480881.2019.1603608.

Mytilinaios I, Salih M, Schofield HK, Lambert RJW. Growth curve prediction from optical density data. Int J Food Microbiol. 2012;154(3). doi: 10.1016/j.ijfoodmicro.2011.12.035.

Ngece K, Ntondini TL, Khwaza V, Paca AM, Aderibigbe BA. Polyene-based derivatives with antifungal activities. Pharmaceutics. 2024;16(8). doi: 10.3390/pharmaceutics16081065.

Núñez Pons L, Calcinai B, Gates RD. Who’s there? – First morphological and DNA barcoding catalogue of the shallow Hawai’ian sponge fauna. PLoS One. 2017;12(12):e0189357. doi: 10.1371/journal.pone.0189357.

Fiehn O. Metabolomics by gas chromatography-mass spectrometry: The combination of targeted and untargeted profiling. Curr Protoc Mol Biol. 2017;7(3).

Pallela R, Janapala VR. Comparative ultrastructural and biochemical studies of four demosponges from Gulf of Mannar, India. Int J Mar Sci. 2013. doi: 10.5376/ijms.2013.03.0037.

Parvekar P, Palaskar J, Metgud S, Maria R, Dutta S. The minimum inhibitory concentration (MIC) and minimum bactericidal concentration (MBC) of silver nanoparticles against Staphylococcus aureus. Biomater Investig Dent. 2020;7(1). doi: 10.1080/26415275.2020.1796674.

Patantis G, Rahmadara G, Elfidasari D, Chasanah E. Molecular identification of sponges obtained from Seribu Islands National Park and their associated bacteria. Squalen Bull Mar Fish Postharvest Biotechnol. 2014;8. doi: 10.15578/squalen.v8i1.80.

Puah PY, Herng Lee DJ, Mak KH, Ang HJ, Chen HC, Moh PY, et al. Extractable impurities from fluoropolymer-based membrane filters—interference in high-throughput, untargeted analysis. RSC Adv. 2019;9(55). doi: 10.1039/c9ra06198c.

Rahman NIA, Ramzi MM, Rawi NN, Siong JYF, Bakar K, Bhubalan K, et al. Characterization of antibiofilm compound from marine sponge Stylissa carteri. Environ Sci Pollut Res. 2024;31(25):37552–37563. doi: 10.1007/s11356-024-33704-0.

Ramya AK, Sethumadhavan K. A comparative study of GCMS analysis of bioactive compound isolated from marine algae-derived endophytic fungi. J Pharm Technol Clin Pharmacol. 2022;30:482–490. doi: 10.53555/jptcp.v30i17.2439.

Richardson JP. Candida albicans: A major fungal pathogen of humans. Pathogens. 2022;11(4). doi: 10.3390/pathogens11040459.

Salam MA, Al-Amin MY, Pawar JS, Akhter N, Lucy IB. Conventional methods and future trends in antimicrobial susceptibility testing. Saudi J Biol Sci. 2023;30(3). doi: 10.1016/j.sjbs.2023.103582.

Salli K, Lehtinen MJ, Tiihonen K, Ouwehand AC. Xylitol's health benefits beyond dental health: A comprehensive review. Nutrients. 2019;11(8). doi: 10.3390/nu11081813.

Sandes J, Muricy G, Cavalcanti FF, Pinheiro U, Annunziata BB. Biodiversity of Porifera in Brazil. Zoologia (Curitiba). 2024;41. doi: 10.1590/s1984-4689.v41.e24017.

Santi Martignago CC, de Souza Barbosa C, Garcia Motta H, Soares-Silva B, Maso Lopes Peres EP, et al. Exploring antibacterial properties of marine sponge-derived natural compounds: A systematic review. Mar Drugs. 2025;23(1). doi: 10.3390/md23010043.

Schöttner S, Hoffmann F, Cárdenas P, Rapp HT, Boetius A, Ramette A. Relationships between host phylogeny, host type, and bacterial community diversity in cold-water coral reef sponges. PLoS One. 2013;8(2). doi: 10.1371/journal.pone.0055505.

SenGupta DJ, Cookson BT. SeqSharp: A general approach for improving cycle-sequencing that facilitates a robust one-step combined amplification and sequencing method. J Mol Diagn. 2010;12(3). doi: 10.2353/jmoldx.2010.090134.

Sharma S, Srivastava R, Srivastava A, Maurya PK, Chandra P. Biomedical potential of marine sponges. In: Marine Sponges: Chemicobiological and Biomedical Applications. 2016. doi: 10.1007/978-81-322-2794-6_16.

Sharma T, Kavlekar D, Ponnapakkam L. Marine drugs from sponge-microbe association—A review. Mar Drugs. 2010;8:1417–1468. doi: 10.3390/md8041417.

Sugrani A, Natsir H, Djide MN, Ahmad A. Antibacterial and anticancer activity of protein sponges collected from the waters of Kapoposang Island of South Sulawesi, Indonesia. Int Res J Pharm. 2019;10(1). doi: 10.7897/2230-8407.100115.

Timmers MA, Vicente J, Webb M, Jury CP, Toonen RJ. Sponging up diversity: Evaluating metabarcoding performance for a taxonomically challenging phylum within a complex cryptobenthic community. Environ DNA. 2022;4(1). doi: 10.1002/edn3.163.

Tuttle AR, Trahan ND, Son MS. Growth and maintenance of Escherichia coli laboratory strains. Curr Protoc. 2021;1(1). doi: 10.1002/cpz1.20.

Valdez CA, Leif RN, Sanner RD, Corzett TH, Dreyer ML, Mason KE. Structural modification of fentanyls for their retrospective identification by gas chromatographic analysis using chloroformate chemistry. Sci Rep. 2021;11(1). doi: 10.1038/s41598-021-01896-x.

van Soest R, Manconi R, Boury-Esnault N, Hooper JA, Rützler K, de Voogd NJ, et al. WPD: World Porifera Database. 2019. doi: 10.14284/359.

Varijakzhan D, Loh JY, Yap WS, Yusoff K, Seboussi R, Lim SHE, et al. Bioactive compounds from marine sponges: Fundamentals and applications. Mar Drugs. 2021;19(5). doi: 10.3390/md19050246.

Williams SE, Stennett HL, Back CR, Tiwari K, Gomez JO, Challand MR, et al. The Bristol sponge microbiome collection: A unique repository of deep-sea microorganisms and associated natural products. Antibiotics. 2020;9(8). doi: 10.3390/antibiotics9080509.

Wright AE, Killday KB, Chakrabarti D, Guzmán EA, Harmody D, McCarthy PJ, et al. Dragmacidin G, a bioactive bis-indole alkaloid from a deep-water sponge of the genus Spongosorites. Mar Drugs. 2017;15(1). doi: 10.3390/md15010016.

Yu HB, Liu XF, Xu Y, Gan JH, Jiao WH, Shen Y, Lin HW. Woodylides A-C, new cytotoxic linear polyketides from the South China Sea sponge Plakortis simplex. Mar Drugs. 2012;10(5). doi: 10.3390/md10051027.

Zhang D, Kan X, Huss S, Jiang L, Chen LQ, Hu Y. Using phylogenetic analysis to investigate eukaryotic gene origin. J Vis Exp. 2018. doi: 10.3791/56684.

Zou Y, Zhang Z, Zeng Y, Hu H, Hao Y, Huang S, Li B. Common methods for phylogenetic tree construction and their implementation in R. Bioengineering. 2024;11(5). doi: 10.3390/bioengineering11050480.

figures and tables Click here to access/download;Figure;Figures and Tables.docx

FIGURES

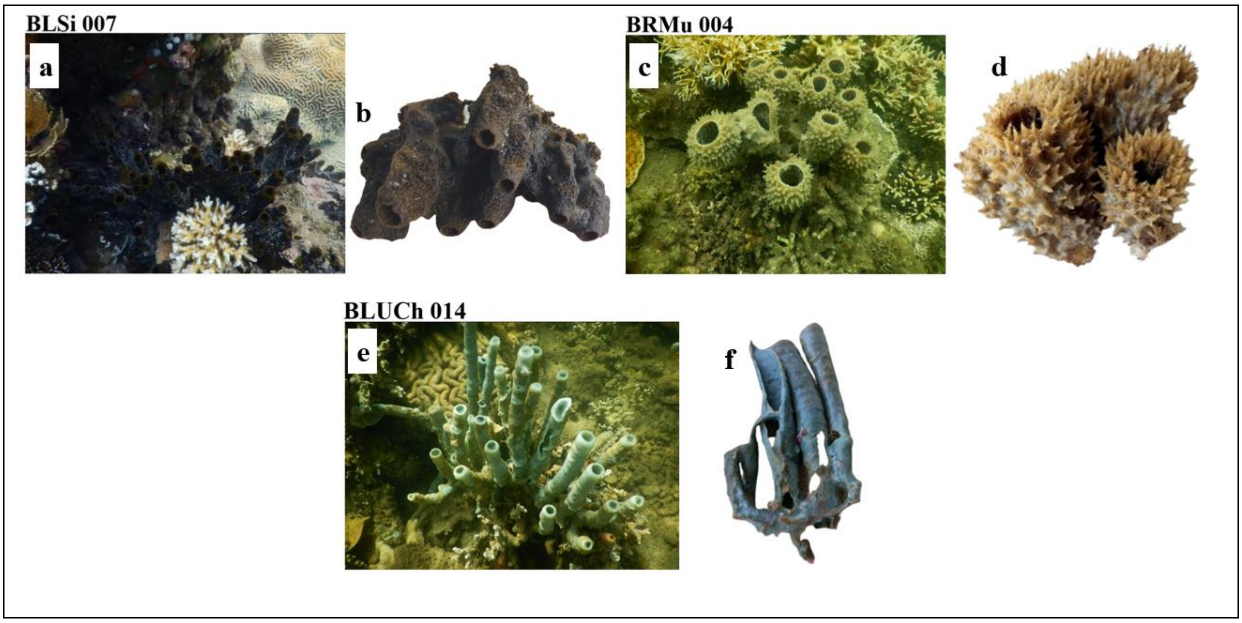

Figure 1: Photographic documentation of marine sponges from the Kenyan coastline, presented as follows: **(a)** *Biemna fistulosa* (Voucher specimen (BLSi 007) (in-situ) **(b)** *B. fistulosa* (detached) **(c)** *Callyspongia diffusa* (Voucher specimen BRMu 004) (in-situ) **(d)** *C. diffusa* (detached) **(e)** *Haliclona fascigera* (Voucher specimen BLUCh 014) (in-situ) **(f)** *Haliclona fascigera* (detached).

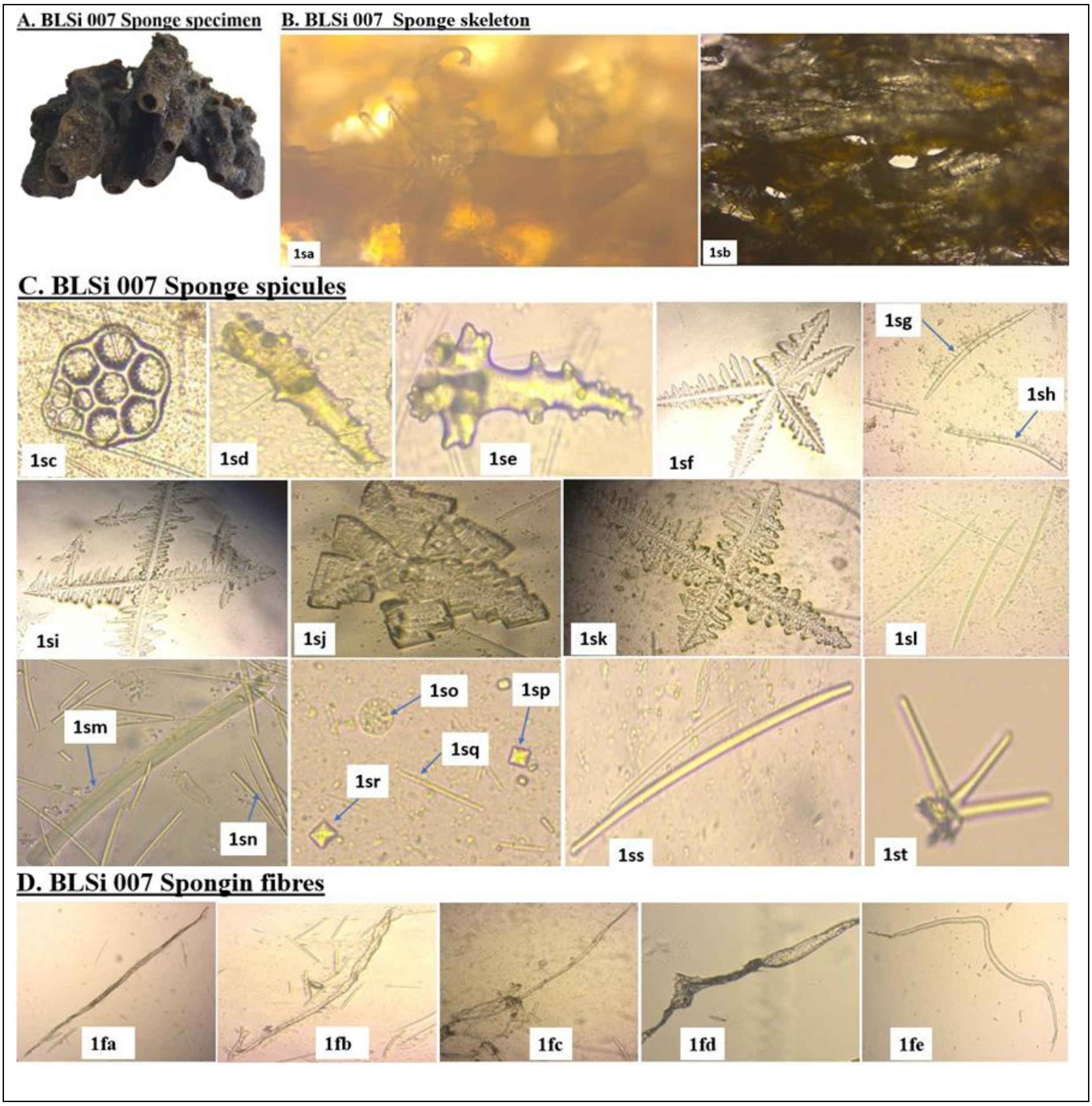

Figure 2: A. **Marine poriferan BLSi 007**, *Biemna fistulosa*; **B. sponge skeleton**: *(1sa):* perpendicular section; and *(1sb):* a tangential section (40x magnification); **C. sponge spicules:** *(1sc):* synapta plates; *(1sd)* and *(1se):* megascleres acanthostyles; *(1sf):* pentactines megascleres with digits at the tentacles; *(1sg):* curved oxeas; *(1sh):* curved styles; *(1si)* and *(1sk):* stauractines megascleres with digits at the tentacles; *(1sj):* pentactines megascleres with digits at the tentacles; *(1sl):* styles; *(1sm):* tabulated strongyles; *(1sn):* raphides; *(1so):* Microbiota (*Coscinodiscus radiatus*); *(1sp)* and *(1sr):* sterrasters; *(1sq):* microstrongyles; *(1ss):* strongyles; and *(1st):* dendroclones (unique to extinct sponge); **D. spongin fibers:** *(1fa):* simple elongated spongin fibers; *(1fb):* simple irregular spongin fibers; *(1fc):* Spongin fiber forming a fiber network on one end; *(1fd):* Spongin fiber with an irregular shape; *(1fe):* spongin fiber with a curved structure.

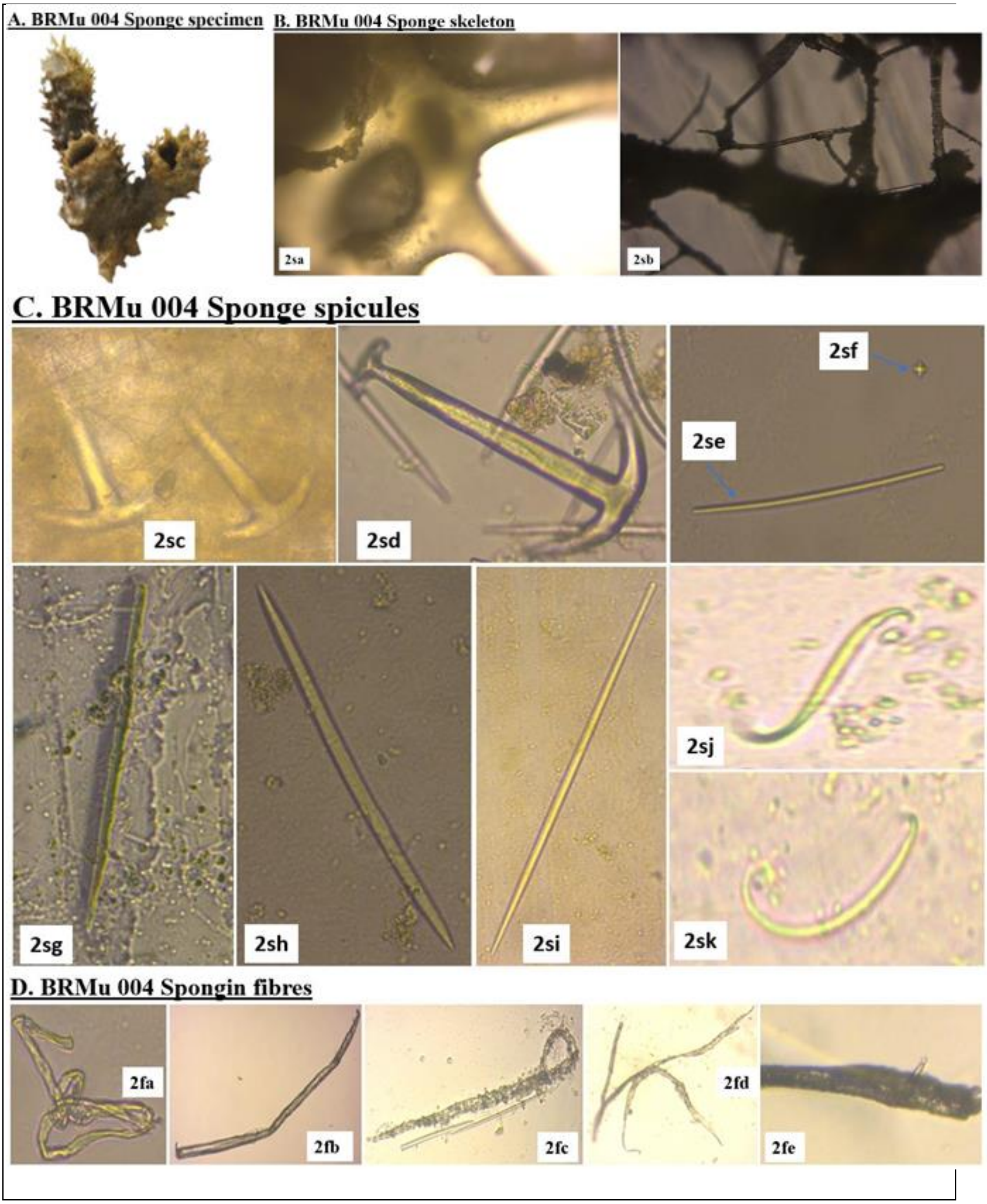

Figure 3: A. **Marine poriferan BRMu 004**, *Callyspongia diffusa*; **B. sponge skeleton**: *(2sa):* perpendicular section; and *(2sb):* a tangential section (40x magnification); **C. sponge spicules:** *(2sc)* and *(2sd):* anchorates; *(2se):* strongyles; *(2sf):* sterrasters; *(2sg):* acanthostyles; *(2sh):* curved oxeas; *(2si):* styles; *(2sj):* S sigmas; and *(2sk):* C sigmas; **D. spongin fibers:** *(2fa):* twisted thick spongin fibers; *(2fb):* Spongin fibers with bent thickened cell walls; *(2fc):* spongin fibers with a complete bent (*Microcoleus vaginatus* attaching on the surface); *(2fd):* spongin fibers with an anastomosing system; and *(2fe):* spongin fibers with hard collagen material (spicules protruding).

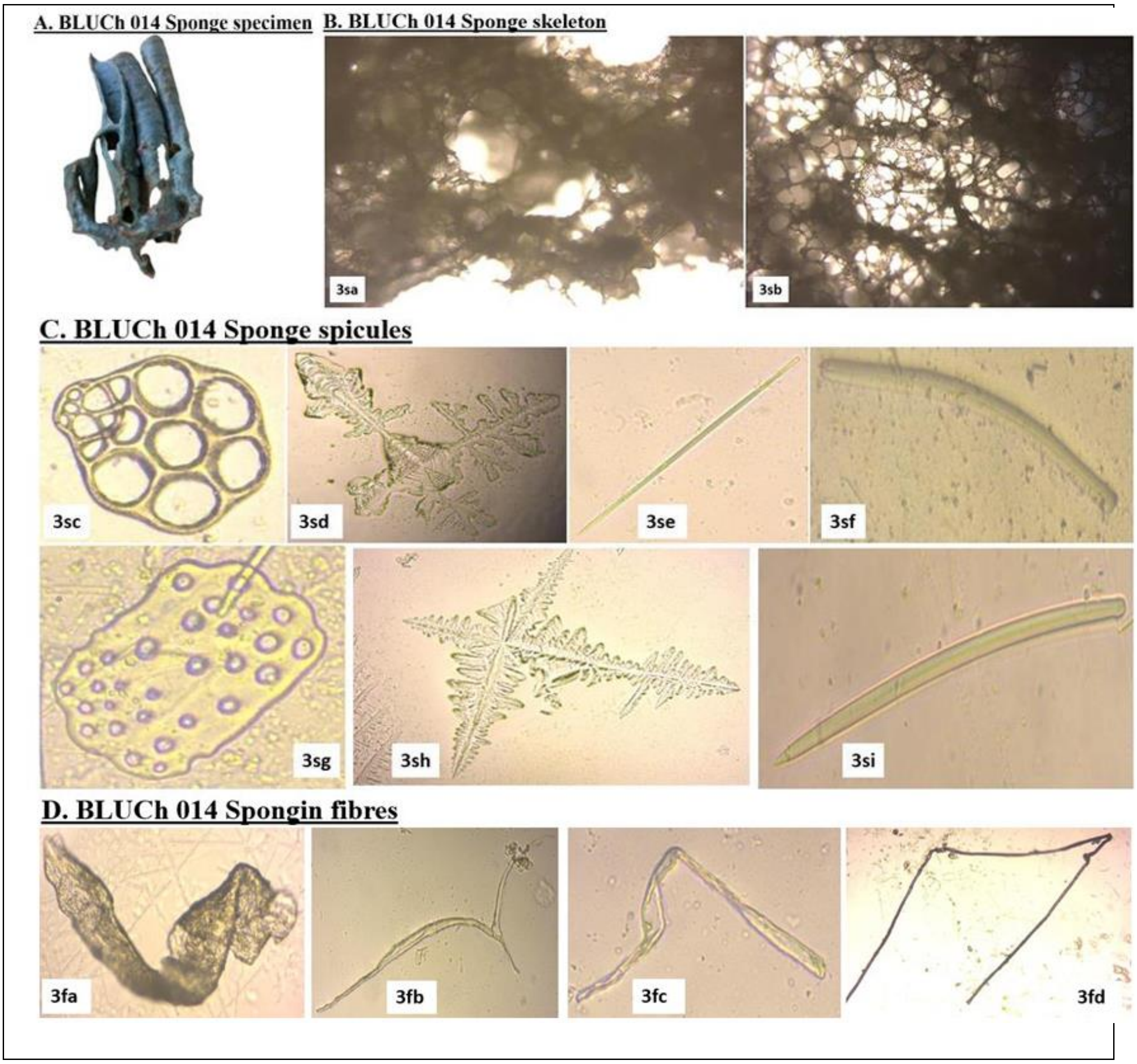

Figure 4: A. Marine poriferan BLUCh 014, *Haliclona fascigera*; **B. sponge skeleton**: *(3sa):* perpendicular section; and *(3sb):* a tangential section (40x magnification); **C. sponge spicules:** *(3sc):* synapta plates; *(3sd)* and *(3sh):* stauractines megascleres with digits at the tentacles; *(3se);* styles; *(3sf):* tuberculated curved strongyles; *(3sg):* large plates of calcareous deposits; *(3si):* styles; **D. spongin fibers:** *(3fa):* spongin fibers with a thick flat structure; *(3fb):* branched spongin fibers; *(3fc):* twisted spongin fibers with an open transparent lumen; *(3fd):* spongin fibers with thickened cell walls and a smooth transparent lumen.

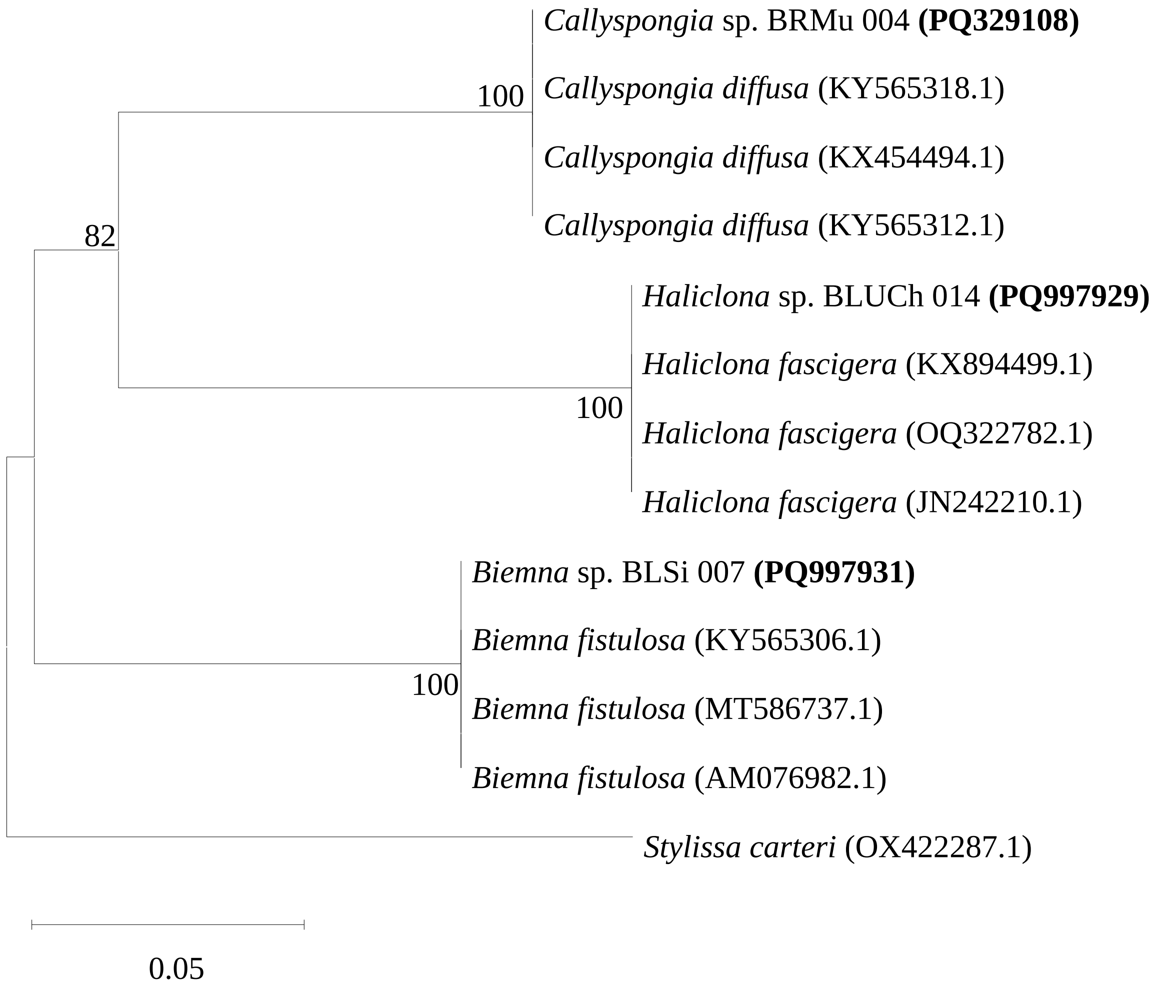

Figure 5: Phylogenetic Relationships of Metazoan CO1 Sequences with Closely Related Sponge Species. The phylogenetic tree was rooted using *Stylissa carteri* (OX422287.1). Bootstrap values exceeding 50%, derived from 1000 replications, are indicated at the branch nodes. The scale bar represents 0.05 substitutions per nucleotide.

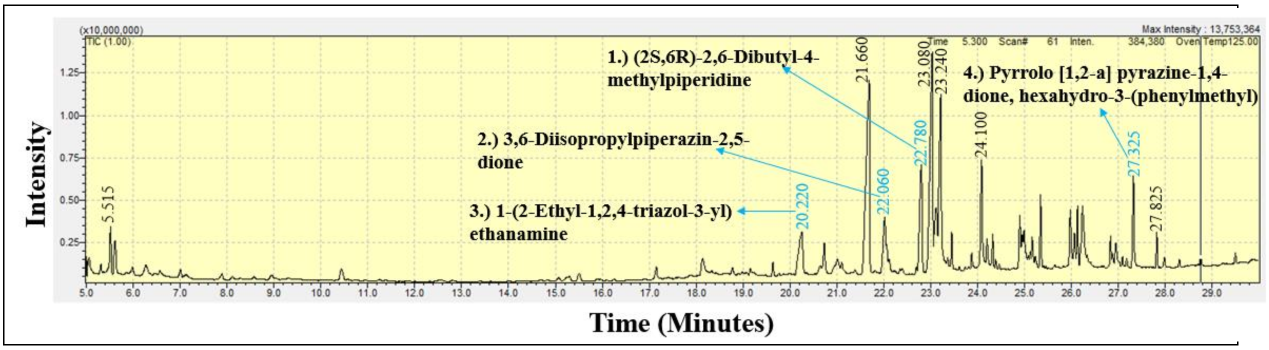

Figure 6: GC-MS chromatogram analysis of the methanolic extract of *Biemna fistulosa* (BLSi 007), highlighting four potent bioactive compounds

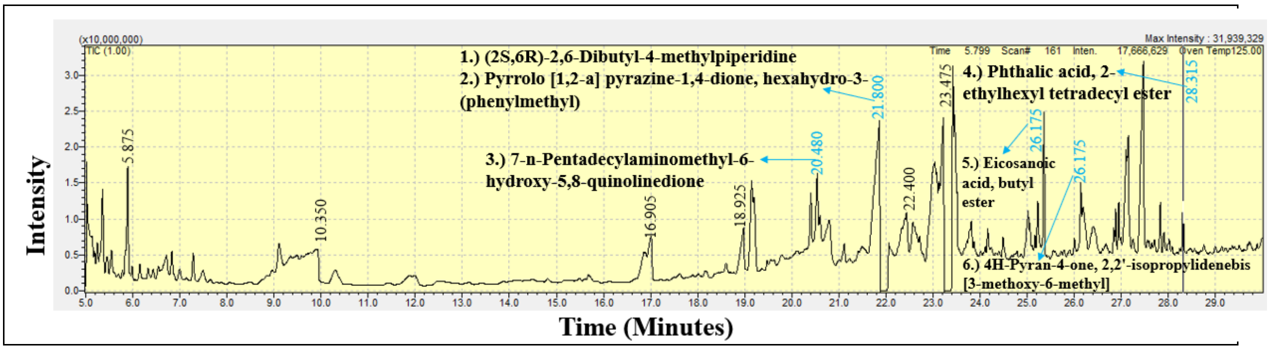

Figure 7: GC-MS chromatogram analysis of the ethyl acetate extract of *Callyspongia diffusa* (BRMu 004), identifying six secondary bioactive compounds.

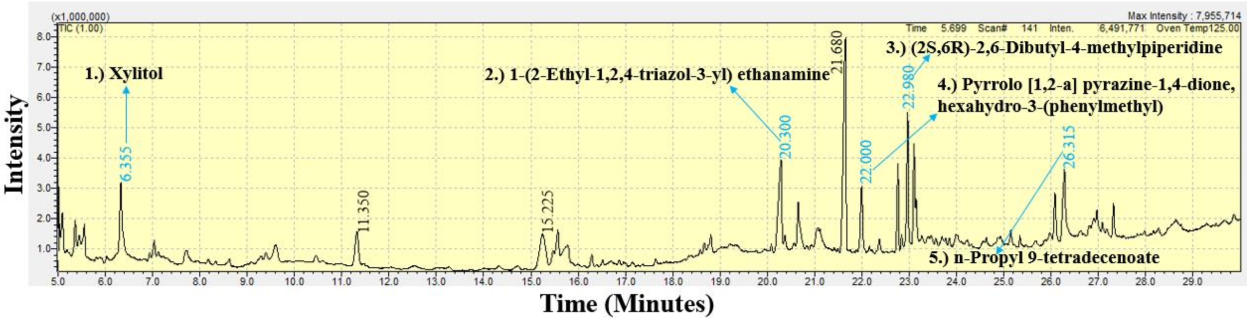

Figure 8: GC-MS chromatogram analysis of the methanolic extract of *Haliclona fascigera* (BLUCh 014), revealing five bioactive compounds.

TABLES

Table 1: Taxonomic affiliation of marine metazoan sponges with their closest phylogenetic relatives

Sample ID	Accession No.	Location	Closest taxonomic affiliation	Isolation Source	Country	% ID
**BLSi 007**	PQ997931	Sii Island (4°40'46.1"S, 39°17'01.9"E)	*Biemna fistulosa* (AM076982.1)	Coral reefs (Núñez Pons et al., 2017)	United States	98
**BRMu 004**	PQ329108	Mundini (4°39'10.0"S, 39°21'41.0"E)	*Callyspongia diffusa* (KX454494.1)	Indo-Pacific region (Pallela & Janapala, 2013)	India	100
**BLUCh 014**	PQ997929	Ras Kiromo (4°38'45.5"S, 39°19'23.4"E)	*Haliclona fascigera* (OQ322782.1)	Island (Handayani et al., 2015)	Indonesia	99

Table 2: Antimicrobial activities of dichloromethane organic crude extracts from the selected marine sponges against the tested human pathogens.

	Dichloromethane extracts inhibition zone diameters

		(mm)			
		**Fungi**	**Gram-negative bacteria**		**Gram-positive bacteria**
**Sample ID**	Sponge species	*Candida Albicans*	*Escherichia coli*	*Pseudomonas aeruginosa*	*Staphylococcus aureus*
**BLSi 007**	*Biemna fistulosa*	0.00 ± 0.0[c]	9.00 ± 1.2[g]	15.33 ± 1.5[cdefg]	13.30 ± 1.5[de]
**BRMu 004**	*Callyspongia diffusa*	0.00 ± 0.0[c]	12.33 ± 0.9[g]	16.00 ± 1.5[g]	0.00 ± 0.0[e]
**BLUCh 014**	*Haliclona fascigera*	0.00 ± 0.0[c]	8.00 ± 0.6[g]	7.67 ± 0.7[efg]	9.33 ± 0.9[de]
	**Positive control**	29.33 ± 1.5[ab]	31.67 ± 0.7[ab]	31.22 ± 0.6[ab]	31.11 ± 0.2[ab]
	**Negative control**	0.00 ± 0.0[c]	0.00 ± 0.0 [g]	0.00 ± 0.0[g]	0.00 ± 0.0[e]

Table 3: Antimicrobial activities of methanolic organic crude extracts from the selected marine sponges against the tested human pathogens.

		Methanolic extracts inhibition zone diameters (mm)			
		**Fungi**	**Gram-negative bacteria**		**Gram-positive bacteria**
**Sample ID**	Sponge species	*Candida Albicans*	*Escherichia coli*	*Pseudomonas aeruginosa*	*Staphylococcus aureus*
**BLSi 007**	*Biemna fistulosa*	15.67 ± 1.2[abc]	28.00 ± 3.5[abcd]	19.67 ± 1.2[abc]	25.33 ± 0.9[abc]
**BRMu 004**	*Callyspongia diffusa*	9.67 ± 2.2[cd]	0.00 ± 0.0[d]	0.00 ± 0.0[c]	0.00 ± 0.0[d]
**BLUCh 014**	*Haliclona fascigera*	10.33 ± 0.9[abc]	28.33 ± 3.8[abcd]	15.67 ± 0.7[ab]	24.67 ± 1.2[abc]
	**Positive control**	25.67 ± 1.2[ab]	27.67 ± 0.9[a]	26.67 ± 0.7[ab]	31.11 ± 0.2[abc]
	**Negative control**	0.00 ± 0.0[d]	0.00 ± 0.0[d]	0.00 ± 0.0[c]	0.00 ± 0.0[d]

Table 4: Antimicrobial activities of ethyl acetate organic crude extracts from the selected marine sponges against the tested human pathogens.

		Ethyl acetate extracts inhibition zone diameters (mm)			
		**Fungi**	**Gram-negative bacteria**		**Gram-positive bacteria**

Sample ID	Sponge species	*Candida Albicans*	*Escherichia coli*	*Pseudomonas aeruginosa*	*Staphylococcus aureus*
**BLSi 007**	*Biemna fistulosa*	0.00 ± 0.0^{c}	8.33 ± 0.3^{cd}	0.00 ± 0.0^{e}	0.00 ± 0.0^{d}
**BRMu 004**	*Callyspongia diffusa*	0.00 ± 0.0^{c}	13.00 ± 2.1^{d}	29.33 ± 2.4^{cde}	9.33 ± 0.9^{d}
**BLUCh 014**	*Haliclona fascigera*	0.00 ± 0.0^{c}	8.67 ± 1.2^{cd}	8.33 ± 0.3^{e}	24.67 ± 1.2^{cd}
	**Positive control**	25.67 ± 1.2^{ab}	27.67 ± 0.9^{a}	26.67 ± 0.7^{abcd}	31.11 ± 0.2^{abc}
	**Negative control**	0.00 ± 0.0^{c}	0.00 ± 0.0^{d}	0.00 ± 0.0^{e}	0.00 ± 0.0^{d}

Table 5: Minimum inhibitory concentrations (MIC) of dichloromethane, methanolic and ethyl acetate organic crude extracts of the selected marine sponges against the tested human pathogens.

			**MIC (mg/ml)**	
**Sample ID**	**Marine sponge**	**Extract**	***Escherichia coli***	***Pseudomonas aeruginosa***
**BLSi 007**	*Biemna fistulosa*	Methanolic	2.46 ± 0.01^{a}	-
**BRMu 004**	*Callyspongia diffusa*	Ethyl acetate	-	2.63 ± 0.01^{a}
**BLD 014**	*Haliclona fascigera*		0.53 ± 0.01^{a}	-
		**Positive Control**	1.36 ± 0.00^{a}	1.94 ± 0.00^{a}
		**Negative Control**	0.00 ± 0.00	0.00 ± 0.00

Table 6: Classification of marine sponge compounds identified via GC-MS in organic extracts from *Biemna fistulosa*, *Callyspongia diffusa*, and *Haliclona fascigera* collected from Kenyan waters.

No.	***Compound chemical class***	***Marine sponge compounds***
1.	α-Ketoglutaric acids	2-Oxopentanedioic acid
2.	Alkenes	1-Dodecene
3.	Alkynes	2-Octyne, 1,1-diethoxy
4.	Amide ester derivatives	L-Proline, N-valeryl-, pentadecyl ester,
5.	Amines	Tris(dimethylamino)methane, 2-Propanamine, 2-methyl-N-(phenylmethylene)-, N-oxide, Propanamide, 2,2-dimethyl-N-(2,6-dimethylphenyl), 1,4-Pentanediamine, N1, N1-diethyl
6.	Amino acid derivatives	l-Proline, N-allyloxycarbonyl-, propyl ester, L-Proline, N-valeryl-, hexadecyl ester, L-Proline, N-valeryl-, tetradecyl ester, l-Leucine, N-cyclopropylcarbonyl-, pentadecyl ester, l-Leucine, N-cyclopropylcarbonyl-, undecyl ester, 3-Pyrrolidin-2-yl-propionic acid
7.	Aromatic hydrocarbons	Azulene, Naphthalene, 1H-Indene, 1-methylene, Benzene, 1,3-diethyl-5-methyl, Cyclopropanemethanol, 1-phenyl-, 2-Isopropylbenzaldehyde, Benzene, 2,4-diethyl-1-methyl-, Benzene, 1,4-diethyl-2-methyl-, Benzene, 1-ethyl-3-(1-methylethyl)-, Benzene, 1,3-dimethyl-5-(1-methylethyl)-, Benzene, 2,4-diethyl-1-methyl-, 3,4-Dimethylcumene
8.	Bicyclic chemical compounds	Isosorbide,
9.	Carboxylic acid anhydrides	2-Dodecen-1-yl (-) succinic anhydride, 3-Heptenoic acid
10.	Chiral amino alcohols	2-Pyrrolidinemethanol, 2-methyl-, (S)
11.	Cyclic alcohols	Bicyclo [3.1.1] heptan-3-ol, 2,6,6-trimethyl-, [1R-(1. alpha.,2. beta.,3. alpha.,5. alpha.)]
12.	Cyclic alkanes	Cyclobutane, 2-hexyl-1,1,4-trimethyl-, cis, Cyclopropane, 1-methyl-1-(1-methylethyl)-2-nonyl
13.	Cyclic amines	diketone
14.	Cyclic dipeptides	3,6-Diisopropylpiperazin-2,5-dione
15.	Diazabicyclo compounds	3-Methyl-1,4-diazabicyclo [4.3.0] nonan-2,5-dione, N-acetyl
16.	Epoxides	Oxirane, 2-methyl-2-(1-methylpropyl)
17.	Ergot alkaloids	Ergotamine, Dihydroergotamine
18.	Esterified fatty acids	Octadecanoic acid, 2-(2-hydroxyethoxy) ethyl ester, Dodecanoic acid, 2-octyl, trans-9-Octadecenoic acid, pentyl ester
19.	Esterified form of ascorbic acid (vitamin C)	l-(+)-Ascorbic acid 2,6-dihexadecanoate
20.	Fatty acids	Decanoic acid, 10-(2-hexylcyclopropyl), Eicosanoic acid, Pentadecanoic acid, Octanoic acid, 7-oxo, Palmitoleic acid, cis-Vaccenic acid, 9-Hexadecenoic acid, cis-10-Nonadecenoic acid, cis-10-Heptadecenoic acid, 9-Eicosenoic acid, (Z)
21.	Fatty alcohols	1-Dodecanol

22.	Heterocyclic boron-containing compound	1,3,2-Dioxaborinane, 2,4-diethyl-5-methyl-6-propyl
23.	Heterocyclic organic compounds	Pyrrolo[1,2-a] pyrazine-1,4-dione, hexahydro-3-(2-methylpropyl), Hexahydro-2H-pyrido(1,2-a) pyrazin-3(4H)-one, Octahydro-1H-pyrido(1,2-c) pyrimidin-1-one, 5,6,7,8-Tetrahydrofurazano[4,5-c] azepin-4-one, 3-[(3,3-Dimethyl-1-azetidinyl) imino]-2,2-dimethyl-1-propanol, 3-Furazancarboxamide, 4-(1-aziridinyl), N-Methyl-9-aza-tricyclo [6.2.2.0(2,7)] dodec-2,4,6,11-tetraene-10-one, 1,3-Dioxolane, 2,4,5-trimethyl, 2-t-Butyl-5-propyl- [1,3] dioxolan-4-one, 1,3-Dioxolane, 4,5-dimethyl-2-pentadecyl, Piperidine, 2-(tetrahydro-2-furanyl), Pyrrolidine-5-one, 2-[3-hydroxypropyl], 2-Pyrrolidinemethanol, 1-methyl, 2-t-Butyl-6-chloromethyl- [1,3] dioxan-4-one, 2-Piperazinimine, 1,3,3,4-tetramethyl-N-(methylsulfonyl), 4H-Pyran-4-one, 2,2'-isopropylidenebis [3-methoxy-6-methyl, 5-Isopropenyl-3,3-dimethyl-dihydrofuran-2-one, (2R,5S)-2,6,10,10-Tetramethyl-1-oxaspiro [4.5] decan-7-one, Pyrrolo [1,2-a] pyrazine-1,4-dione, hexahydro-3-(phenylmethyl), Ergotaman-3',6',18-trione, 9,10-dihydro-12'-hydroxy-2'-methyl-5'-(phenylmethyl)-, (5’. alpha.,10. alpha.), 6,7-Dihydro-5H-pyrrolo [2,1-c] [1,2,4] triazole-3-carboxylic acid, 3-Pyrrolidin-2-yl-propionic acid, Piperazine-3,5-dione, 1-tetradecanoyl, 2-Benzimidazolinethione, hexahydro, 2,5-Methanopyrano[3,2-b] pyrrole, hexahydro-1-methyl
24.	Hydrazones	4-Heptanone, dimethylhydrazone
25.	Imidazoles	1H-Imidazole, 1-methyl
26.	Ketals	2-Propanone, 1,1,3,3-tetrabutoxy
27.	Ketones	2-Cyclohexen-1-one, 3-methyl, 2-Cyclohexen-1-one, 6-(1-hydroxy-1-methylethyl)-3-methyl
28.	Organic esters	Butanoic acid, 4-chloro-4-oxo-, ethyl ester, Butanoic acid, 2-(hydroxymethyl)-, ethyl ester, (R), Succinic acid, butyl pentyl ester, Butanedioic acid, dibutyl ester, Succinic acid, butyl hexyl ester, 2-Pyrrolidinecarboxylic acid-5-oxo-, ethyl ester, Pivalate, (3-nitrobicyclo [2.2.1] hept-5-en-2-yl) methyl ester, Acetic acid, 2-propylpentyl ester, Acetic acid, chloro-, decyl ester, Cyclohexanecarboxylic acid, 4-propyl-, 4-cyanophenyl ester, trans
29.	Organic nitrogenous compounds	Histamine
30.	Phthalic acid esters	Phthalic acid, 5-methylhex-2-yl pentadecyl ester, Phthalic acid, 5-methylhex-2-yl heptadecyl ester, Phthalic acid, 5-methylhex-2-yl hexadecyl ester, Phthalic acid, butyl undecyl ester, Phthalic acid, 2-ethylhexyl tetradecyl ester
31.	Polycyclic hydrocarbon	[4.2.2] Propella-2,4,7,9-tetraene
32.	Protected sugar derivative	Ribofuranose, 1,5-anhydro-2,3-O-isopropylidene-, d
33.	Pyrazole derivatives	1H-Pyrazole-1-carboxaldehyde, 4-ethyl-4,5-dihydro-5-propyl
34.	Quinolinedione derivatives	7-n-Pentadecylaminomethyl-6-hydroxy-5,8-quinolinedione
35.	Spiro compounds	1-Oxaspiro [4.4] nonan-4-one, 2-isopropyl
36.	Sugar alcohols	1,3:2,5-Dimethylene-4-methyl-d-rhamnitol, 1,3:2,5-Dimethylene-l-rhamnitol, D-Arabinitol, Xylitol, 6-Desoxy-l-gulitol

37.	Synthetic compounds	Deoxyspergualin
38.	Triazole derivatives	1-(2-Ethyl-1,2,4-triazol-3-yl) ethanamine
39.	Triglycerides	9-Octadecenoic acid, 1,2,3-propanetriyl ester, (E, E, E)

Table 7: Characteristics and antimicrobial activity of selected sponges' natural products identified in the GC-MS analysis of *Biemna fistulosa, Callyspongia diffusa,* and *Haliclona fascigera* organic extracts.

Sponge extract source	Type of compounds	Retention Time (Minutes)	Compound	Hit spectrum and chemical structure of the bioactive compounds	Molecular formula	Molecular Weight (g/mol)	Quality of similarity (%)	Bioactivity
Biemna fistulosa, Callyspongia diffusa and *Haliclona fascigera*	Cyclic amines	22.780	(2S,6R)-2,6-Dibutyl-4-methylpiperidine	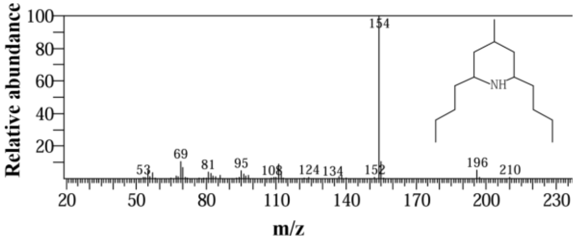	$C_{14}H_{29}N$	211	82	Antimicrobial activity; inhibits topoisomerase II (DNA gyrase) and topoisomerase IV (Ramya & Sethumadhavan, 2022)
Biemna fistulosa, Callyspongia diffusa and *Haliclona fascigera*	Pyrazole derivative	22.780	Pyrrolo[1,2-a] pyrazine-1,4-dione; hexahydro-3-(2-methylpropyl)	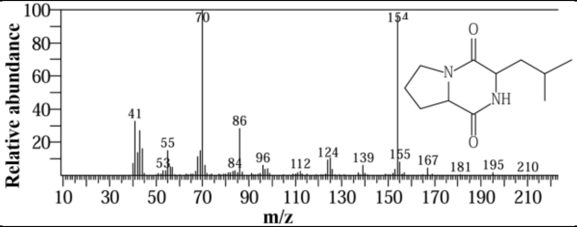	$C_{11}H_{18}N_2O_2$	210	86	Antifungal (Al-Askar et al., 2024); antioxidant and antibacterial activity (Kiran et al., 2018).
Biemna fistulosa and *Callyspongia diffusa*	Pyrazole derivative	27.325	Pyrrolo [1,2-a] pyrazine-1,4-dione, hexahydro-3-(phenylmethyl)	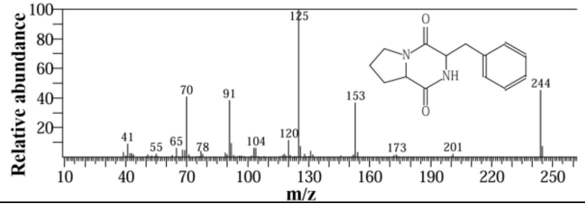	$C_{14}H_{16}N_2O_2$	244	85	

Biemna fistulosa, and *Haliclona fascigera*	Heterocyclic amine	20.220	1-(2-Ethyl-1,2,4-triazol-3-yl) ethanamine	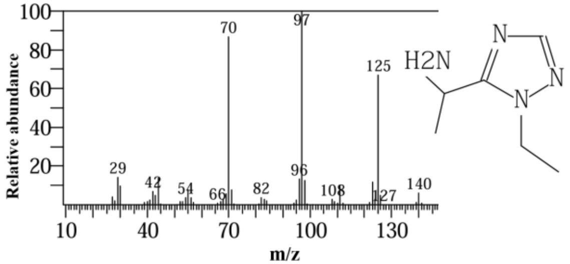	$C_6H_{12}N_4$	140	78	Pharmacological activities such as enzyme inhibition, antifungal, anticancer and antibacterial properties (Gupta et al., 2023)
Biemna fistulosa, and *Haliclona fascigera*	L-Proline derivatives	22.780	L-Proline, N-valeryl-, heptadecyl ester	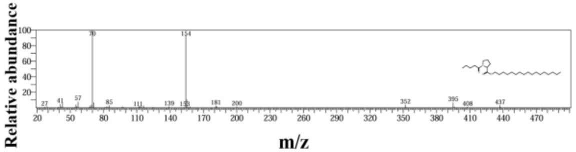	$C_{27}H_{51}NO_3$	437	80	Antiviral activity in plants (Abdelkhalek et al., 2022) and Antitumor agents (Bach & Takagi, 2013)
Biemna fistulosa	Diketopiperazines	22.060	3,6-Diisopropylpiperazin-2,5-dione	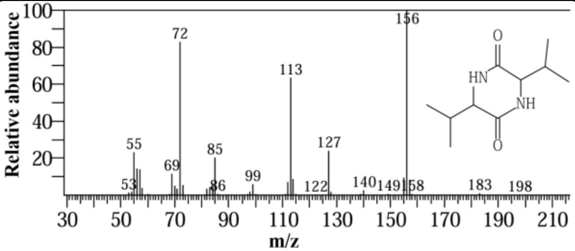	$C_{10}H_{18}N_2O_2$	198	77	Biological activities, including enzyme inhibition and antimicrobial properties (Jia et al., 2022)
Biemna fistulosa	Diketopiperazines	22.060	Piperazine-3,5-dione, 1-tetradecanoyl	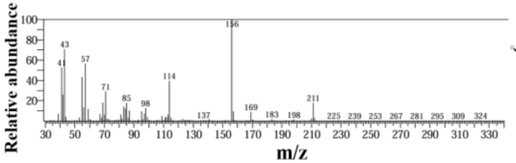	$C_{18}H_{32}N_2O_3$	324	76	
Biemna fistulosa	Fatty acids	27.825	Decanoic acid, 10-(2-hexylcyclopropyl)	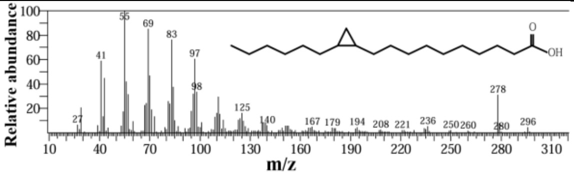	$C_{19}H_{36}O_2$	296	84	Used in identifying bacteria and in research for studying its role in bacterial cell membrane protection and metabolism (Casillas-Vargas et al., 2021)
Callyspongia diffusa	Quinolinedione derivative	20.480	7-n-Pentadecylaminomethyl-6-hydroxy-5,8-quinolinedione	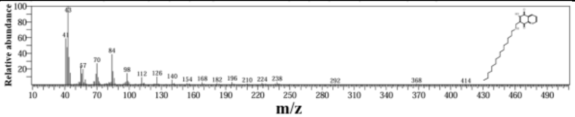	$C_{25}H_{38}N_2O_3$	414	75	Antibacterial; antifungal; antimalarial; anticancer and anti-inflammatory (Kadela-Tomanek et al., 2019)

Callyspongia diffusa	Pyrazole derivative	26.175	4H-Pyran-4-one, 2,2'-isopropylidene bis [3-methoxy-6-methyl]	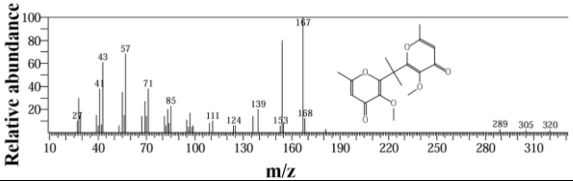	$C_{17}H_{20}O_6$	320	74	Antibiotic, anti-inflammatory, antimalarial, antimicrobial, antiviral, anti-diabetic, anti-tumor, herbicidal, insecticidal, and analgesic activities (Almalki, 2023)
Callyspongia diffusa	Lipophilic chemicals (phthalate acid esters)	28.315	Phthalic acid, 2-ethylhexyl tetradecyl ester	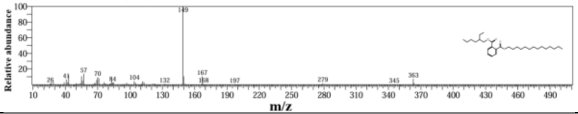	$C_{30}H_{50}O_4$	474	87	Antimicrobial, insecticidal, allelopathic and other biological activities (Huang et al., 2021)
Callyspongia diffusa	Triglyceride (Triolein)	27.830	9-Octadecenoic acid, 1,2,3-propanetriyl ester, (E, E, E)	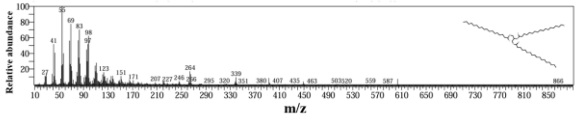	$C_{57}H_{104}O_6$	884	83	Pharmaceuticals as a carrier or excipient in drug formulations; and an emollient and moisturizing therapy in skin care products; (N. Kumar & Sharma, 2023).
Haliclona fascigera	Sugar alcohols	6.355	Xylitol	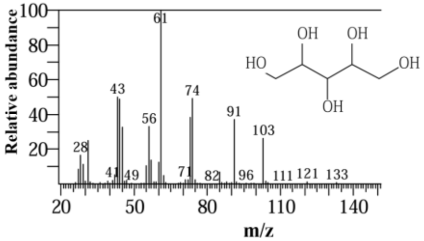	$C_5H_{12}O_5$	152	75	Antibacterial, diabetes management, ear infection treatment, and gut health (Gasmi Benahmed et al., 2020); antimicrobial, dental and respiratory health (Salli et al., 2019); and anticancer, anti-inflammation and bone health (Ahuja et al., 2020);
Haliclona fascigera	Alkyl esters	26.315	n-Propyl 9-tetradecenoate	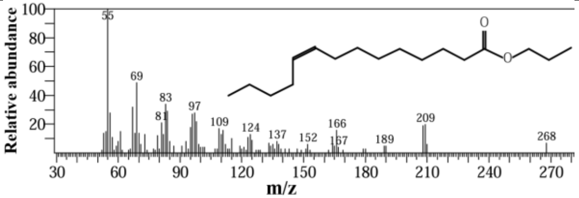	$C_{17}H_{32}O_2$	268	72	Antifungal activity against *Candida albicans* (Kadhim et al., 2016)
Haliclona fascigera	Esters Ergot alkaloids	11.350	Dodecanoic acid, 2-penten-1-yl ester	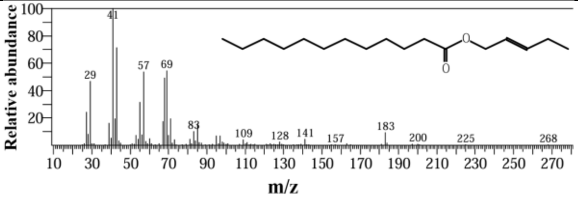	$C_{17}H_{32}O_2$	268	78	Anticancer, antioxidant, and antimicrobial activities (Hamouda et al., 2024)

Click here to access/download
Supporting Information
ASSESSMENT OF SPONGE BIODIVERSITY PERMISSION.pdf
