# Supplementary material for: Characterization and bioactivity potential of marine sponges (Biemna fistulosa, Callyspongia diffusa, and Haliclona fascigera) from Kenyan coastal waters
Source: PLoS One. 2025 Jul 24;20(7):e0325642. doi: 10.1371/journal.pone.0325642 (PMC12289071; doi:10.1371/journal.pone.0325642)
Supplement: Attachment — Submitted filename: Response to Reviewers.docx [file pone.0325642.s006.docx]

The Editor-in-Chief,

PLOS ONE.

Dear Sir/Madam,

23^rd^ June 2025

Rebuttal Letter

Article: "**Characterization and Bioactivity Potential of Marine Sponges (*Biemna fistulosa, Callyspongia diffusa*, and *Haliclona fascigera*) from Kenyan Coastal Waters**"

Dear academic editor and reviewers,

We sincerely appreciate the time and effort the editor and reviewers have devoted to evaluating our manuscript, as well as their valuable insights. In the sections that follow, we provide a detailed explanation of how we have addressed each point they raised. For clarity, all comments from the editor and reviewers are presented in bold font. All manuscript text has been italicized, with revisions highlighted in yellow and all responses to the editor and reviewers are in blue colour.

**Journal Requirements:**

Comments to the Author:

**When submitting your revision, we need you to address these additional requirements.**

1. **Please ensure that your manuscript meets PLOS ONE's style requirements, including those for file naming. The PLOS ONE style templates can be found at**

**https://journals.plos.org/plosone/s/file?id=wjVg/PLOSOne_formatting_sample_main_body.pdf and**

**https://journals.plos.org/plosone/s/file?id=ba62/PLOSOne_formatting_sample_title_authors_affil iations.pdf**

Response: We adapted the manuscript to meet the style requirements.

Comments to the Author:

**2. In your Methods section, please provide additional information regarding the permits you obtained for the work. Please ensure you have included the full name of the authority that approved the field site access and, if no permits were required, a brief statement explaining why.**

Response: The material and methods section included the following details in the manuscript:

## *Ethical statement*

*This research study was officially authorized by the National Commission for Science, Technology and Innovation of Kenya (NACOSTI) under License No: NACOSTI/P/25/4174598. Ethical approval for the study was granted by the Technical University of Mombasa (TUM) under approval No: TUM SERC PhD/006/2025, ensuring that all research activities adhered to institutional ethical standards.*

*Additionally, the Kenya Marine and Fisheries Research Institute (KMFRI) provided the necessary access permit for field sampling under Reference No: GOK_PC Target C82 39-1/22, facilitating compliance with regulatory requirements for environmental research. Furthermore, the fieldwork did not involve any endangered or protected species, aligning with conservation guidelines and ethical research principles.*

Copies of the relevant authorization documents mentioned above will be uploaded as supplementary materials for reference.

Comments to the Author:

**3. Thank you for stating the following financial disclosure:**

**“We sincerely thank the Kenya Marine and Fisheries Research Institute (KMFRI) for their invaluable support in partially funding for the research (GOK-PC Target C82 39-1).”Please state what role the funders took in the study.  If the funders had no role, please state: "****The funders had no role in study design, data collection and analysis, decision to publish, or preparation of the manuscript."**

**If this statement is not correct, you must amend it as needed.**

**Please include this amended Role of Funder statement** **in your cover letter; we will change the online submission form on your behalf.**

Response: The following information was removed from the funding section in the manuscript:

*Funding*

*We sincerely thank the Kenya Marine and Fisheries Research Institute (KMFRI) for their invaluable support (GOK-PC Target C82 39-1).*

In the cover letter, the statement below was added.

*‘‘The funders had no role in study design, data collection and analysis, decision to publish, or preparation of the manuscript.’’*

Comments to the Author:

**4. Thank you for stating the following in the Funding Section of your manuscript:**

**“We sincerely thank the Kenya Marine and Fisheries Research Institute (KMFRI) for their invaluable support (GOK-PC Target C82 39-1).”**

**We note that you have provided funding information that is currently declared in your Funding Statement. However, funding information should not appear in the Acknowledgments section or other areas of your manuscript. We will only publish funding information present in the Funding Statement section of the online submission form.**

**Please remove any funding-related text from the manuscript and let us know how you would like to update your Funding Statement. Currently, your Funding Statement reads as follows:**

**“We sincerely thank the Kenya Marine and Fisheries Research Institute (KMFRI) for their invaluable support in partially funding for the research (GOK-PC Target C82 39-1).”Please state what role the funders took in the study.  If the funders had no role, please state: "The funders had no role in study design, data collection and analysis, decision to publish, or preparation of the manuscript."**

**Please include your amended statements within your cover letter; we will change the online submission form on your behalf.**

Response: The following information was removed from the funding section in the manuscript:

*Funding*

*We sincerely thank the Kenya Marine and Fisheries Research Institute (KMFRI) for their invaluable support (GOK-PC Target C82 39-1).*

The acknowledgment section in the manuscript was revised as follows (Line 707–709):

# *Acknowledgements*

*We sincerely thank the Kenya Marine and Fisheries Research Institute (KMFRI) and the Technical University of Mombasa (TUM) for their invaluable contributions.*

Comments to the Author:

**5. We note that Figures 1, 2, 3, and 4 in your submission contain copyrighted images. All PLOS content is published under the Creative Commons Attribution License (CC BY 4.0), which means that the manuscript, images, and Supporting Information files will be freely available online, and any third party is permitted to access, download, copy, distribute, and use these materials in any way, even commercially, with proper attribution. For more information, see our copyright guidelines:** [**http://journals.plos.org/plosone/s/licenses-and-copyright**](http://journals.plos.org/plosone/s/licenses-and-copyright)**.**

**We require you to either (1) present written permission from the copyright holder to publish these figures specifically under the CC BY 4.0 license, or (2) remove the figures from your submission:**

**1. You may seek permission from the original copyright holder of Figures 1, 2, 3, and 4 to publish the content specifically under the CC BY 4.0 license.**

**We recommend that you contact the original copyright holder with the Content Permission Form (**[**http://journals.plos.org/plosone/s/file?id=7c09/content-permission-form.pdf**](http://journals.plos.org/plosone/s/file?id=7c09/content-permission-form.pdf)**) and the following text:**

**“I request permission for the open-access journal PLOS ONE to publish XXX under the Creative Commons Attribution License (CCAL) CC BY 4.0 (**[**http://creativecommons.org/licenses/by/4.0/**](http://creativecommons.org/licenses/by/4.0/)**). Please be aware that this license allows unrestricted use and distribution, even commercially, by third parties. Please reply and provide explicit written permission to publish XXX under a CC BY license and complete the attached form.”**

**Please upload the completed Content Permission Form or other proof of granted permissions as an "Other" file with your submission.**

**In the figure caption of the copyrighted figure, please include the following text: “Reprinted from [ref] under a CC BY license, with permission from [name of publisher], original copyright [original copyright year].”**

**2. If you are unable to obtain permission from the original copyright holder to publish these figures under the CC BY 4.0 license or if the copyright holder’s requirements are incompatible with the CC BY 4.0 license, please either i) remove the figure or ii) supply a replacement figure that complies with the CC BY 4.0 license. Please check copyright information on all replacement figures and update the figure caption with source information. If applicable, please specify in the figure caption text when a figure is similar but not identical to the original image and is therefore for illustrative purposes only.**

Response: Thank you for reaching out regarding Figures 1, 2, 3, and 4 in our submission.

We would like to clarify that these figures were originally produced by the corresponding author (Teresia Nyambura Wacira), who captured the microscopic images using a Primo Star ZEISS image analyzer microscope. The microphotographs were taken with an Axio Cam ERc5s digital camera (Carl Zeiss, Germany). Given that these images were created entirely by the author and do not originate from copyrighted sources, we confirm that they do not require third-party permissions for publication under the Creative Commons Attribution License (CC BY 4.0).

Please let us know if further documentation is required to verify the originality of these figures. We appreciate your guidance and look forward to resolving this matter.

The image captions in the results section of the manuscript have been updated to include ‘‘(Source: Author),’’ providing the following details (Line 347–396):

***Fig 1:*** *Photographic documentation of marine sponges from the Kenyan coastline, presented as follows:* ***(a)*** *Biemna fistulosa (Voucher specimen (BLSi 007) (in-situ)* ***(b)*** *B. fistulosa (detached)* ***(c)*** *Callyspongia diffusa (Voucher specimen BRMu 004) (in-situ)* ***(d)*** *C. diffusa (detached)* ***(e)*** *Haliclona fascigera (Voucher specimen* *BLUCh 014) (in-situ)* ***(f)*** *Haliclona fascigera (detached)* *(Source: Author).*

*Fig 2: A. Marine poriferan BLSi 007, Biemna fistulosa; B. sponge skeleton: (1sa): perpendicular section; and (1sb): a tangential section (40x magnification); C. sponge spicules: (1sc): synapta plates; (1sd) and (1se): megascleres acanthostyles; (1sf): pentactines megascleres with digits at the tentacles; (1sg): curved oxeas; (1sh): curved styles; (1si) and (1sk): stauractines megascleres with digits at the tentacles; (1sj): pentactines megascleres with digits at the tentacles; (1sl): styles; (1sm): tabulated strongyles; (1sn): raphides; (1so): Microbiota (Coscinodiscus radiatus); (1sp) and (1sr): sterrasters; (1sq): microstrongyles; (1ss): strongyles; and (1st): dendroclones (unique to extinct sponge); D. spongin fibers: (1fa): simple elongated spongin fibers; (1fb): simple irregular spongin fibers; (1fc): Spongin fiber forming a fiber network on one end; (1fd): Spongin fiber with an irregular shape; (1fe): spongin fiber with a curved structure (Source: Author).*

*Fig 3: A. Marine poriferan BRMu 004, Callyspongia diffusa; B. sponge skeleton: (2sa): perpendicular section; and (2sb): a tangential section (40x magnification); C. sponge spicules: (2sc) and (2sd): anchorates; (2se): strongyles; (2sf): sterrasters; (2sg): acanthostyles; (2sh): curved oxeas; (2si): styles; (2sj): S sigmas; and (2sk): C sigmas; D. spongin fibers: (2fa): twisted thick spongin fibers; (2fb): Spongin fibers with bent thickened cell walls; (2fc): spongin fibers with a complete bent (Microcoleus vaginatus attaching on the surface); (2fd): spongin fibers with an anastomosing system; and (2fe): spongin fibers with hard collagen material (spicules protruding) (Source: Author).*

*Fig 4: A.* *Marine poriferan BLUCh 014, Haliclona fascigera; B. sponge skeleton: (3sa): perpendicular section; and (3sb): a tangential section (40x magnification); C. sponge spicules: (3sc): synapta plates; (3sd) and (3sh): stauractines megascleres with digits at the tentacles; (3se); styles; (3sf): tuberculated curved strongyles; (3sg): large plates of calcareous deposits; (3si): styles; D. spongin fibers: (3fa): spongin fibers with a thick flat structure; (3fb): branched spongin fibers; (3fc): twisted spongin fibers with an open transparent lumen; (3fd): spongin fibers with thickened cell walls and a smooth transparent lumen* *(Source: Author).*

Comments to the Author:

**Additional Editor Comments:**

**Thank you for submitting your manuscript to PLOS ONE. After careful consideration, we feel that it has merit but does not fully meet PLOS ONE’s publication criteria as it currently stands. Therefore, we invite you to submit a revised version of the manuscript that addresses the points raised during the review process.**

Response: Our detailed answers can be found below.

**Reviewer #1**

Comments to the Author:

**In the financial disclosure, there's missing information, like: Who received the funding? and if the sponsors or funders play any role in this study?.
- Line 13 - 15: It's not necessary to rewrite the name in "Corresponding Author"; simply enter an asterisk (*) followed by the email address. Please review PLOS Affiliations Guidelines.
- Line 17 - 20: Lots of information about the authors, I believe that data is stored internally when the article is submitted. Please review PLOS Affiliations Guidelines.
-Line 164 - 166: Important to indicate the conditions under which the purified products were shipped.**

Response: Clarification on Funding Details

Thank you for reaching out regarding the financial disclosure in our manuscript.

We would like to clarify that the corresponding author received funding specifically for sampling activities and DNA sequencing services. This support facilitated the collection and genetic analysis of sponge specimens included in the study.

In the cover letter, the statement below was added.

*‘‘The funders had no role in study design, data collection and analysis, decision to publish, or preparation of the manuscript.’’*

The corrected version, following the PLOS Affiliations Guidelines for Line 13–18, was updated to:

*Corresponding Author* Email: tnyambura@kmfri.go.ke*

Based on the PLOS Affiliations Guidelines, here’s the corrected version as we revised in the manuscript:

*Authors:*

*Huxley M. Makonde*

*Joseph K. Nyingi*

*Christopher M. Aura*

*Cromwell M. Kibiti*

The contact details have been removed, as such information is typically stored internally upon submission.

A revised version of the manuscript incorporated the shipping conditions (Line 199–201):

*The purified products were preserved under dry ice conditions and then shipped to Inqaba Biotec, a commercial sequencing service provider in South Africa, for Sanger sequencing.*

**Reviewer #2**

Comments to the Author:

**All my comments have been included in the reviewed document. I suggest including ethical considerations in the manuscript and the permissions for collecting the sponges. The revision of each part of the document is indicated in the manuscript.**

Response: The material and methods section included the following details in the manuscript (Line 114–124):

*Ethical statement*

*This research study was officially authorized by the National Commission for Science, Technology and Innovation of Kenya (NACOSTI) under License No: NACOSTI/P/25/4174598. Ethical approval for the study was granted by the Technical University of Mombasa (TUM) under approval No: TUM SERC PhD/006/2025, ensuring that all research activities adhered to institutional ethical standards.*

*Additionally, the Kenya Marine and Fisheries Research Institute (KMFRI) provided the necessary access permit for field sampling under Reference No: GOK_PC Target C82 39-1/22, facilitating compliance with regulatory requirements for environmental research. Furthermore, the fieldwork did not involve any endangered or protected species, aligning with conservation guidelines and ethical research principles.*

Copies of the relevant authorization documents mentioned above will be uploaded as supplementary materials for reference.

Comments to the Author:

**Which species of porifera had more antibacterial activity?**

Response: In the reviewed manuscript, the discussion (Line 642–645) and in the conclusion (Line 690–692) section responded to this question:

*Additionally, the H. fascigera extract exhibited the highest antibacterial activity among the tested sponge species.*

*Furthermore, the methanolic extract of H. fascigera demonstrated the most potent antibacterial and antifungal activity among the marine sponge extracts evaluated.*

Comments to the Author:

**What about the results on Staphylococcus aureus and *candida albicans*?**

Response: In the reviewed manuscript, the results section responded to this question (Line 441–446):

*B. fistulosa exhibited the strongest antibacterial activity against S. aureus in methanolic extracts (25.33 ± 0.9 mm), whereas H. fascigera showed the highest potency in ethyl acetate extracts (24.67 ± 1.2 mm). Despite their notable inhibitory potential, both sponges were less effective than the standard antibiotic, streptomycin (31.11 ± 0.2 mm) (Table 4). Methanolic extracts of B. fistulosa exhibited a higher antifungal activity (15.67 ± 1.2mm) against C. albicans compared to the methanolic extract of H. fascigera (10.33 ± 0.9 mm) and C. diffusa (9.67 ± 2.2 mm) (Table 5).*

Comments to the Author:

**This reference is not connected with a reference.**

Response: The reference was correctly positioned within the appropriate sentence in the introduction section of the manuscript (Line 77–80):

*Furthermore, while various sponge-derived compounds are recognized for their antibacterial and antifungal activities, there is inadequate literature and comprehensive data on the identification and bioactivity studies of marine sponges from the Kenyan coastal waters [15].*

Comments to the Author:

**Which is the diversity of Kenya**

Response: In the reviewed manuscript, the introduction section responded to this question (Line 103–107):

*The World Porifera Database recognizes over 9,700 sponge species globally, with many belonging to the class Demospongiae, which includes species found in Kenya. Additionally, a study identified Axinella infundibuliformis from the Kenyan coast, reporting its antimicrobial activity against Staphylococcus aureus and Pseudomonas aeruginosa [21].*

Comments to the Author:

**The aim must be specific and not generic**

Response: In the reviewed manuscript, the introduction section was revised as per the reviewer’s guidance (Line 107–111):

*This study aimed to address the scientific gap by identifying and characterizing three marine sponge species, Biemna fistulosa, Callyspongia diffusa, and Haliclona fascigera from Kenyan coastal waters. Their bioactive potential was assessed through antimicrobial susceptibility testing against Escherichia coli, Pseudomonas aeruginosa, Staphylococcus aureus, and Candida albicans.*

Comments to the Author:

**What about this species???**

Response: The correct species name was accurately documented in the results section of the manuscript (Line 354–355):

*The species, B. fistulosa, typically inhabited sandy shores and mangrove lagoons.*

Comments to the Author:

**Appears to be methods, not results**

Response: The methodology-related sentences were removed from the results section and moved to the methodology section of the manuscript (Line 158–162):

*The marine sponges were coded based on their lifeform color and the study site of collection. The prefixes BL, BR, and BLU denoted black, brown, and blue sponges, respectively. These were followed by Si, Mu, and Ch, representing Sii Island, Mundini, and Ras Kiromo, respectively, along with the specimen number assigned to each sponge from the Kenyan coastline.*

The other information was captured in the data analysis section of the manuscript (Line 330–335):

*The data were recorded in triplicate and presented as mean ± standard deviation (SD) for the inhibition zones. Statistical tests of means sharing identical superscripts (*) within the column were considered not significantly different from one another, as determined through Fisher's Least Significant Difference (LSD) test at a 95% confidence level, following a post hoc analysis conducted using Minitab software version 21.4.1. Differences were classified as statistically significant at P < 0.05 (α = 0.05).*

In the discussion section, the necessary corrections were made according to the reviewer's recommendations (Line 541–542):

*The morphological identification of B. fistulosa, C. diffusa, and H. fascigera offers valuable insights into their taxonomic classification and ecological adaptations.*

Comments to the Author:

**Join!!**

Response: The previously separated sentences were merged in the discussion section of the manuscript to enhance clarity and coherence, as required (Line 656–658):

*Elsewhere, [94] revealed that the organic sponge extracts exhibited greater antibacterial activity than the standard antibiotic against S. aureus and E. coli.*

**Reviewer #3**

Comments to the Author:

**1. Summary of the research**

**Wacira et al. used morphological and genetic analyses to identify three sponge species and tested their extracts against human pathogens E. coli, P. aeruginosa, S. aureus, and C. albicans. In particular, the authors measured inhibition zone diameters and determined the minimum inhibitory concentrations for each. Furthermore, the authors identified the chemical compounds present. The authors conclude that all three species of sponge showed antimicrobial activity against at least one of the pathogens. Specifically, methanolic extracts from B. fistulosa and H. fascigera had larger inhibition zone diameters for E. coli than the positive control, while ethyl acetate extracts of C. diffusa had a larger inhibition zone diameter for P. aeruginosa than the positive control. While methanolic extracts from B. fistulosa had the largest inhibition zone diameter of all treatments for C. albicans, none were greater than the positive control. H. fascigera had the lowest MBC against E. coli, even lower than the positive control. This study addresses an important topic and provides valuable insights into bioactivity potential of marine sponges. However, I believe rearranging the structure of how the results are presented will improve readability, and expanding on certain details in the introduction and discussion would be necessary to provide adequate context for the study. I recommend the below changes.**

Comments to the Author:

**2. Major issues**

**(1) Further context needed in introduction**

**• Line 82: What are the gaps in knowledge? This paragraph could be expanded i.e. discuss if there are challenges with identifying sponges with morphological techniques, advantages/disadvantages of extract types (methanolic, ethyl acetate..), is there anything else unknown about chemical compounds? If the gap is mainly from the geographic location, perhaps consider including further site details such as oceanography or current bioactive compounds work/industry from the location. I thought the paragraph beginning on line 66 provided good context with examples of chemical compounds with recorded bioactive properties**

Response: The paragraph on knowledge gaps was expanded in the introduction section to incorporate the reviewers' suggestions (Line 81–98):

*Morphological identification of marine sponges is constrained by phenotypic plasticity, environmental variability, and the presence of cryptic species, while DNA barcoding has enhanced taxonomic resolution but remains hindered by incomplete molecular databases for Kenyan coastal sponges [16]. Extraction methodologies significantly influence compound recovery, with methanolic extracts facilitating the isolation of polar bioactive molecules such as alkaloids and flavonoids, albeit with potential thermolabile degradation, ethyl acetate extracts exhibiting superior antimicrobial activity yet limited in their ability to retain highly polar compounds, and dichloromethane extracts preferentially isolating non-polar sterols and terpenoids but demonstrating reduced efficacy against hydrophilic pathogens [17].*

*Although GC-MS analysis has identified numerous distinct compounds, their pharmacological properties, toxicity profiles, and mechanisms of action remain largely unexplored, necessitating further investigation to elucidate their bioactive potential, while oceanographic factors such as seasonal monsoons, nutrient upwelling, and coral reef ecosystems may serve as critical determinants influencing sponge-associated microbial diversity and secondary metabolite production [18]. Moreover, the commercial application of sponge-derived bioactive compounds within Kenya is currently underdeveloped, emphasizing the imperative for interdisciplinary collaborations between marine biotechnology researchers and pharmaceutical industries to facilitate the translation of these bioactive molecules into viable therapeutic agents.*

Comments to the Author:

**(2) Results were somewhat hard to follow starting with section “In Vitro antibiotic and antifungal activity of the marine sponge crude extracts”**

**• Since the text generally discusses results by pathogen and this appears to be the main focus of the manuscript, I suggest to reorganize Tables 2-4 so that each pathogen is one table with the extract types by column and sponges still by row. I believe this will be easier for the reader to see the main points**

Response: (In the figures document and Line 436-453 in the manuscript)

*Table 2: Antimicrobial activities of organic crude extracts from the selected marine sponges against Escherichia coli.*

|  | | *Marine sponge extracts*  *(mm)* | | |
| --- | --- | --- | --- | --- |
| *Sample ID* | *Sponge Species* | *Dichloromethane* | *Methanolic* | *Ethyl Acetate* |
| *BLSi 007* | *Biemna fistulosa* | *9.00 ± 1.2^g^* | *28.00 ± 3.5^abcd^* | *8.33 ± 0.3c^d^* |
| *BRMu 004* | *Callyspongia diffusa* | *12.33 ± 0.9^g^* | *0.00 ± 0.0^d^* | *13.00 ± 2.1^d^* |
| *BLUCh 014* | *Haliclona fascigera* | *8.00 ± 0.6^g^* | *28.33 ± 3.8^abcd^* | *8.67 ± 1.2^cd^* |
| *Positive control* | | *31.67 ± 0.7^ab^* | *27.67 ± 0.9^a^* | *27.67 ± 0.9^a^* |
| *Negative* | | *0.00 ± 0.0 ^g^* | *0.00 ± 0.0^d^* | *0.00±0.0^d^* |

*Table 3: Antimicrobial activities of organic crude extracts from the selected marine sponges against Pseudomonas aeruginosa.*

|  | | *Marine sponge extracts*  *(mm)* | | |
| --- | --- | --- | --- | --- |
| *Sample ID* | *Sponge Species* | *Dichloromethane* | *Methanolic* | *Ethyl Acetate* |
| *BLSi 007* | *Biemna fistulosa* | *15.33 ± 1.5^cdefg^* | *19.67 ± 1.2^abc^* | *0.00 ± 0.0^e^* |
| *BRMu 004* | *Callyspongia diffusa* | *16.00 ± 1.5^g^* | *0.00 ± 0.0^c^* | *29.33 ± 2.4^cde^* |
| *BLUCh 014* | *Haliclona fascigera* | *7.67 ± 0.7^efg^* | *15.67 ± 0.7^ab^* | *8.33 ± 0.3^e^* |
| *Positive control* | | *31.22 ± 0.6^ab^* | *26.67 ± 0.7^ab^* | *26.67 ± 0.7^abcd^* |
| *Negative* | | *0.00 ± 0.0^g^* | *0.00 ± 0.0^c^* | *0.00±0.0^e^* |

*Table 4: Antimicrobial activities of organic crude extracts from the selected marine sponges against Staphylococcus aureus.*

|  | | *Marine sponge extracts*  *(mm)* | | |
| --- | --- | --- | --- | --- |
| *Sample ID* | *Sponge Species* | *Dichloromethane* | *Methanolic* | *Ethyl Acetate* |
| *BLSi 007* | *Biemna fistulosa* | *13.30 ± 1.5^de^* | *25.33 ± 0.9^abc^* | *0.00 ± 0.0^d^* |
| *BRMu 004* | *Callyspongia diffusa* | *0.00 ± 0.0^e^* | *0.00 ± 0.0^d^* | *9.33 ± 0.9^d^* |
| *BLUCh 014* | *Haliclona fascigera* | *9.33 ± 0.9^de^* | *24.67 ± 1.2^abc^* | *24.67 ± 1.2^cd^* |
| *Positive control* | | *31.11 ± 0.2^ab^* | *31.11 ± 0.2^abc^* | *31.11 ± 0.2^abc^* |
| *Negative* | | *0.00 ± 0.0^e^* | *0.00 ± 0.0^d^* | *0.00±0.0^d^* |

*Table 5: Antimicrobial activities of organic crude extracts from the selected marine sponges against Candida albicans.*

|  | | *Marine sponge extracts*  *(mm)* | | |
| --- | --- | --- | --- | --- |
| *Sample ID* | *Sponge Species* | *Dichloromethane* | *Methanolic* | *Ethyl Acetate* |
| *BLSi 007* | *Biemna fistulosa* | *0.00 ± 0.0^c^* | *15.67 ± 1.2^abc^* | *0.00 ± 0.0^c^* |
| *BRMu 004* | *Callyspongia diffusa* | *0.00 ± 0.0^c^* | *9.67 ± 2.2^cd^* | *0.00 ± 0.0^c^* |
| *BLUCh 014* | *Haliclona fascigera* | *0.00 ± 0.0^c^* | *10.33 ± 0.9^abc^* | *0.00 ± 0.0^c^* |
| *Positive control* | | *29.33 ± 1.5^ab^* | *25.67 ± 1.2^ab^* | *25.67 ± 1.2^ab^* |
| *Negative* | | *0.00 ± 0.0^c^* | *0.00 ± 0.0^d^* | *0.00±0.0^c^* |

Comments to the Author:

**• I believe the paragraph beginning on line 419 is repeated several times. I think it should either start on line 403, before "All three [..]" or go in the table captions**

Response: The methodology-related sentences were removed from the results section and moved to the methodology section of the manuscript (Line 158–162):

*The marine sponges were coded based on their lifeform color and the study site of collection. The prefixes BL, BR, and BLU denoted black, brown, and blue sponges, respectively. These were followed by Si, Mu, and Ch, representing Sii Island, Mundini, and Ras Kiromo, respectively, along with the specimen number assigned to each sponge from the Kenyan coastline.*

The other information was captured in the data analysis section of the manuscript (Line 330–335):

*The data were recorded in triplicate and presented as mean ± standard deviation (SD) for the inhibition zones. Statistical tests of means sharing identical superscripts (*) within the column were considered not significantly different from one another, as determined through Fisher's Least Significant Difference (LSD) test at a 95% confidence level, following a post hoc analysis conducted using Minitab software version 21.4.1. Differences were classified as statistically significant at P < 0.05 (α = 0.05).*

Comments to the Author:

**• Lines 455-469 are especially hard to follow. Line 455: Streptomycin exhibited lowest compared to what? Does the next sentence contradict this?**

Response: The revised sentence has been adjusted to address the reviewer's question (Line 457–459):

*Streptomycin demonstrated the lowest MIC compared to the sponge extracts, with values of 1.36 mg/ml against E. coli and 1.94 mg/ml against P. aeruginosa (Table 5).*

Comments to the Author:

**Line 459: what reference drugs does this refer to? Where are these values from, are citations needed here?**

Response: The minimum bactericidal concentration (MBC) values presented in this study were derived from experimental data. The following sentence has been revised to directly address the reviewer's question and clarify the findings (Line 461–462):

*In this study, the* *MBC for the standard streptomycin drug was established at 2.5 mg mL^-1^.*

Comments to the Author:

**(3) Discussion needs a bit more context and seems to jump around a bit starting with line 634.**

**• Good context is provided by discussing examples of chemical compounds with bioactivity of these sponges, however further context is needed in some places i.e. lines 556, 574, and 598**

Response: An additional context has been incorporated into lines as recommended (Line 554–568), (Line 586–596) and (Line 615–621).

*The encrusting nature of B. fistulosa may provide a competitive advantage in wave-exposed environments by reducing drag and facilitating firm attachment to substrates. Similarly, the intricate spicule network in H. fascigera supports its survival in sediment-rich coastal habitats, where stability and substrate interaction are essential for persistence. Comparative studies on sponge skeletal morphologies have revealed similar configurations across species belonging to the same genus or geographic region, suggesting potential evolutionary and environmental drivers shaping skeletal development [73].*

*Beyond their structural attributes, sponge spicules and spongin fibers play essential functional roles in microbial associations and chemical defense. The intricate skeletal matrix of sponges harbors diverse microbial symbionts, with spicules possibly serving as microhabitats for bacteria and other microorganisms (Pawlik and McMurray, 2020). Additionally, bioactive compounds associated with sponge skeletons have demonstrated significant antimicrobial properties, contributing to their defensive strategies against potential pathogenic threats. Research has identified bioactive metabolites within sponge extracts exhibiting antibacterial activity against human pathogens, underscoring the ecological and pharmaceutical relevance of these skeletal structures [75].*

*The formation of distinct sub-clusters in the phylogenetic tree highlights the genetic divergence among these sponge specimens, confirming their classification as separate species. This molecular approach not only refines taxonomic resolution but also provides insights into evolutionary relationships within Demospongiae. The integration of DNA barcoding with phylogenetic analysis is particularly valuable in marine sponge research, where morphological plasticity often complicates species identification. Future studies could expand on this by incorporating additional genetic markers and broader taxonomic sampling to further elucidate phylogenetic relationships and species diversity within these genera [78]. A previous study examined species of shallow Hawaiian sponge fauna in the United States and identified* *Biemna fistulosa and* *Callyspongia diffusa using DNA barcoding [79].*

*A similar study identified 212 bioactive compounds from the genus Callyspongia, and 109 molecules were reported to exhibit bioactivity [87]. Additionally, this research established that the ethyl acetate extract derived from C. diffusa demonstrated bactericidal activity against P. aeruginosa, with an MBC value of 2.5 mg mL^-1^. Marine sponge extracts from a study of the waters of Mauritius exhibited low MBC values, indicating that these extracts could serve as a potential approach to traditional bacterial infection management strategies [18].*

Comments to the Author:

**• After discussing morphological and genetic identification, consider ordering paragraphs by result combination rather than methods. I.e. so that methyl acetate extracts of C. diffusa exhibited inhibitory activity against P. aeruginosa is discussed, followed by identified chemical compounds for this, followed by MIC. Then discuss methanolic extracts from B. fistulosa and H. fascigera against E. coli in the same format, etc. (Suggest to move paragraph beginning on line 634 to 613, and paragraph beginning on line 644 to after that one)**

Response: The suggested restructuring has been implemented (Line 615–621) and (Line 642–653)

*A similar study identified 212 bioactive compounds from the genus Callyspongia, and 109 molecules were reported to exhibit bioactivity [87]. Additionally, this research established that the ethyl acetate extract derived from C. diffusa demonstrated bactericidal activity against P. aeruginosa, with an MBC value of 2.5 mg mL^-1^. Marine sponge extracts from a study of the waters of Mauritius exhibited low MBC values, indicating that these extracts could serve as a potential approach to traditional bacterial infection management strategies [18].*

*Additionally, the H. fascigera extract exhibited the highest antibacterial activity among the tested sponge species. It also demonstrated the lowest Minimum Inhibitory Concentration (MIC) against E. coli (0.53 ± 0.01 mg mL^-1^), surpassing the efficacy of streptomycin (1.36 ± 0.00 mg mL^-1^). Furthermore, its Minimum Bactericidal Concentration (MBC) was recorded at 1.25 mg/mL, outperforming streptomycin (2.5 mg mL^-1^). These findings confirm that H. fascigera's methanolic extract possesses superior antibacterial potential compared to the extracts of B. fistulosa and C. diffusa, indicating its promising application in antimicrobial drug development. In a similar study, H. fascigera, collected from Bidong Island, Malaysia, showed positive results in all in vitro biological studies and exhibited higher antibacterial activities compared to other sponges in the study [93]. Another research also revealed that the butanol fraction of Biemna sp. exhibited significant antibacterial activity, with an MIC value of 0.091 mg mL^-1^ against Escherichia coli [94].*

Comments to the Author:

**• Line 660: is H. fascigera meant to be B. fistulosa? I think that is what the results and tables state. This paragraph seems most related to paragraph 614 and should be moved to line 633**

Response: The correction has been made: *H. fascigera* was replaced with *B. fistulosa* to align with the results and tables. Additionally, the paragraph in question has been repositioned as it aligns more coherently with the mentioned paragraph in the discussion (Line 647).

Comments to the Author:

**• Line 661: I am not clear on where this value is from?**

Response: The value of 2.5 mg/mL was confirmed as the minimum fungicidal concentration (MFC) of the methanolic extract of *H. fascigera* following experimental analysis. This finding reflects the concentration at which complete inhibition of fungal growth was observed, providing a critical benchmark for assessing the extract’s potency (Line 474–476)

*The methanolic extract of H. fascigera demonstrated the most potent fungicidal activity, with a minimum fungicidal concentration (MFC) of 2.5 mg mL^-1^.*

Comments to the Author:

**3. Minor issues**

**(1) Line 49: I believe the first two sentences were meant to be one sentence separated with a comma**

Response: The two sentences at line 45-46 have been merged into a single one.

*Despite a record dating back at least the entire Phanerozoic, approximately 600 million years [1], the magnitude of aquatic marine sponge biodiversity is unknown [2].*

Comments to the Author:

**(2) Lines 55 and 56: “class” and “order” I think should be capitalized**

Response: This correction has been made (line 50-52)

*Among the three classes of sponges, class Demospongiae and orders Halichondrida, Poecilosclerida, and Dictyoceratida are the significant sources of bioactive compounds compared to Hexactinellida and Calcarea [5].*

Comments to the Author:

**(3) Line 93: suggest to state “three” species of sponge instead of “some”**

Response: This correction has been made (line 107-109).

*This study aimed to address the scientific gap by identifying and characterizing three marine sponge species, Biemna fistulosa, Callyspongia diffusa, and Haliclona fascigera from Kenyan coastal waters.*

Comments to the Author:

**(4) Line 262: suggest to add “and fungal” between “bacterial growth” for clarity**

Response: This suggestion has been incorporated (line 289-291).

*The minimum inhibitory concentration (MIC) was determined as the lowest concentration of the sponge extract that visibly inhibited bacterial and fungal growth [61].*

Comments to the Author:

**(5) Line 266: For clarity suggest to add that MBC and MFC were determined for those pathogens that showed an inhibitory zone, if that is indeed the case**

Response: This suggestion has been incorporated (line 294-295).

*The minimum bactericidal (MBC) and fungicidal (MFC) concentrations were determined for sponge extracts that exhibited low MICs against the tested microorganisms.*

Comments to the Author:

**(6) Line 302: were the data tested for normal distribution? What were the sample sizes? Also suggest to specify the factors in the ANOVA for clarity**

Response: Prior to the ANOVA, data were tested for normal distribution using the Fisher's Least Significant Difference (LSD) test to ensure validity of the parametric analysis. A two-way analysis of variance (ANOVA) was performed to assess statistically significant differences in the diameters of inhibition zones across a sample of twenty-three marine sponge extracts.

The sentence was revised to (line 328-330).

*A two-way analysis of variance (ANOVA) was performed to determine the presence of statistically significant differences in the diameters of inhibition zones across a sample size of twenty-three marine sponge extracts.*

Comments to the Author:

**(7) Line 319: how long were samples out of water for? Could this have impacted results?**

Response: Sponge samples were exposed to air for approximately 20 to 25 minutes during collection and initial handling before being placed into cooler boxes with ice packs. Although this brief exposure caused noticeable morphological changes such as shrinkage, increased fragility, and color loss the conditions were consistent across all samples. These alterations were taken into account during downstream analyses, and the standardized handling protocol minimized variability, reducing the likelihood of significant impact on the results.

Comments to the Author:

**(8) Line 558: delete “conducted”**

Response: Done. The word “conducted” has been deleted as requested.

Comments to the Author:

**(9) Line 563: delete comma**

Response: Done.

Comments to the Author:

**(10) Line 578: delete “t” in “indicated”**

Response: Done.

Comments to the Author:

**(11) Line 609: provide geographic context of study discussed**

Response: The sentence was revised to (Lines 655-658)

*Another study from Badi Island of Spermonde Archipelago also revealed the antibacterial activities of crude extracts of H. fascigera against three shrimp pathogenic bacteria [48]. In Mauritius, revealed that the organic sponge extracts exhibited greater antibacterial activity than the standard antibiotic against S. aureus and E. coli [94].*

Comments to the Author:

**(12) Line 614: I thought this was good context and the previous paragraph discussing the bacterial pathogens could benefit from a similar level of detail**

Response: Thank you for that thoughtful observation. The restructuring and enhancement of paragraph detail were addressed in response to an earlier comment regarding the organization of results following morphological and genetic identification. Specifically, the paragraphs were reordered to present extract activity, followed by identified compounds and MIC values this structure was applied consistently to both C. diffusa and extracts from B. fistulosa and H. fascigera. As part of this update, the paragraph discussing bacterial pathogens was revised to include more contextual detail, aligning with the level of specificity you appreciated.

The suggested restructuring has been implemented (Lines 615-632) and (Line 639-657).

*A similar study identified 212 bioactive compounds from the genus Callyspongia, and 109 molecules were reported to exhibit bioactivity [87]. Additionally, this research established that the ethyl acetate extract derived from C. diffusa demonstrated bactericidal activity against P. aeruginosa, with an MBC value of 2.5 mg mL^-1^. Marine sponge extracts from a study of the waters of Mauritius exhibited low MBC values, indicating that these extracts could serve as a potential approach to traditional bacterial infection management strategies [18]. Furthermore, in this study, the 9-octadecenoic acid, 1,2,3-propanetriyl ester E, E, E, a triolein compound, was extracted from the ethyl acetate extract of C. diffusa. This compound holds pharmaceutical significance as a carrier or excipient in drug formulations and is also employed as an emollient and moisturizing agent in dermatological products [88]. In a previous study, the bioactive compound 9-Octadecenoic acid, 1,2,3-propanetriyl ester (E, E, E) was discovered in Cassia angustifolia and demonstrated significant antimicrobial activity [89]. This underscores the genus’s significance as a prolific source of secondary metabolites with promising pharmaceutical applications. The findings of this study are consistent with previous research, reinforcing the role of marine sponges in drug discovery and the development of antimicrobial agents. Further studies should prioritize the isolation and characterization of these bioactive compounds to elucidate their mechanisms of action and explore their potential for therapeutic formulation [87].*

*In our study, H. fascigera exhibited broad-spectrum antibacterial activity, with an inhibition zone diameter (IZD) of 28.33 ± 3.8 mm against E. coli. Additionally, the H. fascigera extract exhibited the highest antibacterial activity among the tested sponge species. It also demonstrated the lowest Minimum Inhibitory Concentration (MIC) against E. coli (0.53 ± 0.01 mg mL^-1^), surpassing the efficacy of streptomycin (1.36 ± 0.00 mg mL^-1^). Furthermore, its Minimum Bactericidal Concentration (MBC) was recorded at 1.25 mg/mL, outperforming streptomycin (2.5 mg mL^-1^). These findings confirm that H. fascigera's methanolic extract possesses superior antibacterial potential compared to the extracts of B. fistulosa and C. diffusa, indicating its promising application in antimicrobial drug development. In a similar study, H. fascigera, collected from Bidong Island, Malaysia, showed positive results in all in vitro biological studies and exhibited higher antibacterial activities compared to other sponges in the study [93]. Another research also revealed that the butanol fraction of Biemna sp. exhibited significant antibacterial activity, with an MIC value of 0.091 mg mL^-1^ against Escherichia coli [94].*

*In a related study, extracts from H. fascigera sourced in Indonesia proved effective in inhibiting the growth of S. aureus and E. coli [95]. Another study also revealed the antibacterial activities of crude extracts of H. fascigera against three shrimp pathogenic bacteria [48]. Elsewhere, [94] revealed that the organic sponge extracts exhibited greater antibacterial activity than the standard antibiotic against S. aureus and E. coli.*

Comments to the Author:

**(13) Line 616: add that it was not higher than the positive control here**

Response: The sentence was revised to (Lines 662-664)

*On one hand, B. fistulosa demonstrated more potent bioactive compounds capable of inhibiting C. albicans growth compared to C. diffusa and H. fascigera; however, its activity did not exceed that of the positive control.*

Comments to the Author:

**4. Other points (confidential)**

**I have selected 'yes' in #3 that the authors have provided their data. However I am not sure because the tables in the manuscript only contain summary statistics. I am available to review a revised version.**

Response: The following information was added in the methodology section of the manuscript (Line 138-145):

*A total of 23 marine sponge samples were obtained from six study sites distributed along Kenya’s northern and southern coastlines, representing a wide spectrum of taxa differing in abundance and distribution (S1-3 Tables). For the purposes of this study, three sponge species each reflecting a distinct abundance category were chosen for detailed analysis. These samples were collected from different study sites along the Southern coastline (Sii Island, Mundini, and Ras Kiromo) and included Callyspongia diffusa (high abundance, found at more than four sites), Biemna fistulosa (moderate abundance, observed at two to three sites), and Haliclona fascigera (rare, recorded at a single site).*

The supporting table was saved as a separate file titled 'Supporting Information' and will be uploaded alongside the revised documents.

***S1 Table:*** *Summary of marine sponge species observed in high abundance (≥4 sites) across Kenyan coastal study areas*

| ***Sponge Species (Taxon)*** | ***Morphotype / Color*** | ***Sites*** | ***Habitat Notes*** |
| --- | --- | --- | --- |
| *Carteriospongia foliascens* | *Fan-shaped / Green* | *Kuruwitu, Kanamai, Sii Island, Mtwapa Creek, Mundini and Ras Kiromo* | *Found on mangroves, sandy lagoons, seagrass beds, sandy beaches, and coral reefs* |
| *Callyspongia diffusa* | *Tube-like / Brown* |  | *Common in seagrass beds and coral reef slopes; tolerates turbid shallow waters* |
| *Callyspongia plicifera* | *Tube-like / Orange* |  | *Often found on coral rubble and protected reef flats* |
| *Callyspongia siphonella* | *Tube-like / Cream* |  | *Typically occurs in shallow reef lagoons and sheltered bays* |
| *Haliclona tubifera* | *Encrusting / Green* |  | *Commonly associated with mangrove roots and soft-bottom habitats* |
| *Haliclona oculata* | *Encrusting / Brown* |  | *Attached to rocks and stones on sandy or rocky substrate* |

***S2 Table:*** *Summary of marine sponge species recorded at moderate abundance (2–3 sites) along the Kenyan coastline*

| ***Sponge Species (Taxon)*** | ***Morphotype / Color*** | ***Sites*** | ***Habitat Notes*** |
| --- | --- | --- | --- |
| *Biemna fistulosa* | *Encrusting / Brown* | *Ras Kiromo and Mtwapa Creek* | *Present along sandy shores and in mangrove lagoons* |
| *Tedania charcoti* | *Pear-shaped / Brown-Orange* | *Sii Island, Mtwapa Creek and Ras Kiromo* | *Shallow tropical waters, near mangrove roots and sandy lagoons* |
| *Callyspongia ramosa* | *Tube-like / Orange-Grey* | *Kanamai and Mtwapa Creek* | *Grows on reef crests and within shallow lagoons* |
| *Callyspongia pseudotoxa* | *Tube-like / Cream-Green* | *Kuruwitu, Kanamai and Mtwapa Creek* | *Found on sandy substrates and patch reef systems* |
| *Haliclona implexiformis* | *Encrusting / Purple* |  | *Typically associated with mangrove channels or soft-sediment substrates* |
| *Haliclona stilensis* | *Encrusting / Brown* | *Kuruwitu, Mtwapa Creek and Sii Island* | *Usually encrusts seagrass blades, rubble, or coral fragments* |
| *Haliclona cinerea* | *Encrusting / Purple* | *Kuruwitu, Kanamai, Ras Kiromo* | *Inhabits intertidal rock pools and coral reef flats* |
| *Callyspongia fallax* | *Tube-like / Grey* | *Mtwapa Creek, Mundini* | *Found on shallow sandy bottoms and in mangrove lagoons* |
| *Haliclona laubenfelsi* | *Encrusting / Cream-Green* | *Sii Island, Mundini, Ras Kiromo* | *Found in protected lagoons and among coral assemblages* |
| *Clathria rugosa* | *Fan-shaped / Brown* |  | *Grows on coral reef slopes and rubble zones* |
| *Clathria reinwardti* | *Fan-shaped / Red* |  | *Occurs in reef crevices and shaded lagoon habitats* |
| *Clathria prolifera* | *Fan-shaped / Orange* |  | *Found on rocky reef faces and in tide pools* |
| *Clathria parthena* | *Fan-shaped / Brown* |  | *Typically inhabits shallow coral reefs and rubble beds* |

***S3 Table:*** *Summary of marine sponge species observed at rare abundance (single site) along the Kenyan coastline*

| ***Sponge Species (Taxon)*** | ***Morphotype / Color*** | ***Sites*** | ***Habitat Notes*** |
| --- | --- | --- | --- |
| *Stylissa carteri* | *Fan-shaped / Orange* | *Sii Island* | *Found in mangrove lagoons, coral reef margins, and sandy beach zones* |
| *Agelas cerebrum* | *Tube-like / Brown* | *Mtwapa Creek* | *Typically found in cryptic reef environments, attached to hard substrata* |
| *Haliclona fascigera* | *Tube-like / Blue* | *Ras Kiromo* | *Shallow sandy bottoms of lagoons and coral reef vicinities* |
| *Paratetilla arcifera* | *Pear-shaped / Brown* | *Kanamai* | *Inhabits seagrass beds, shallow sandy lagoons, and sandy beaches* |

Kind regards,

***Name:* Ms. TERESIA NYAMBURA WACIRA**

***Tel:* +254721723213**

***E-mail: tnyambura@kmfri.go.ke***
